# Environmental predictors of SARS-CoV-2 infection incidence in Catalonia (northwestern Mediterranean)

**DOI:** 10.3389/fpubh.2024.1430902

**Published:** 2024-12-05

**Authors:** Jesús Planella-Morató, Josep L. Pelegrí, Marta Martín-Rey, Anna Olivé Abelló, Xavier Vallès, Josep Roca, Carlos Rodrigo, Oriol Estrada, Ignasi Vallès-Casanova

**Affiliations:** ^1^Departament d’Oceanografia Física i Tecnològica, Institut de Ciències del Mar, CSIC, Barcelona, Spain; ^2^Departament de Física, Universitat de Girona, Girona, Spain; ^3^University School of Health and Sport (EUSES), University of Girona, Girona, Spain; ^4^Departamento de Física de la Tierra y Astrofísica, Universidad Complutense de Madrid, Madrid, Spain; ^5^Fundació Lluita contra les Infeccions, Badalona, Spain; ^6^Fundació Institut per la Recerca Germans Trias i Pujol, Badalona, Spain; ^7^Programa de Salut Internacional Institut Català de la Salut (PROSICS), Badalona, Spain; ^8^Epidemiology Unit, Hospital Universitari Germans Trias i Pujol, Institut Català de la Salut, Badalona, Spain; ^9^Department of Pediatrics, Institut de Recerca Germans Trias i Pujol, Badalona, Spain; ^10^Universitat Autònoma de Barcelona (UAB), Barcelona, Spain; ^11^Directorate for Innovation and Interdisciplinary Cooperation, Northern Metropolitan Region from Barcelona, Institut Català de la Salut, Barcelona, Spain; ^12^Hebrew University of Jerusalem, Jerusalem, Israel; ^13^Centro Oceanográfico de Santander, Instituto Español de Oceanografia, IEO-CSIC, Santander, Spain

**Keywords:** environmental sciences, epidemiology, COVID-19, atmospheric variables, weather-infection correlation, virus spread, climate change

## Abstract

Numerous studies have explored whether and how the spread of the SARS-CoV-2, the causative agent of coronavirus disease 2019 (COVID-19), responds to environmental conditions without reaching consistent answers. Sociodemographic factors, such as variable population density and mobility, as well as the lack of effective epidemiological monitoring, make it difficult to establish robust correlations. Here we carry out a regional cross-correlation study between nine atmospheric variables and an infection index (*I_c_*) estimated from standardized positive polymerase chain reaction (PCR) test cases. The correlations and associated time-lags are used to build a linear multiple-regression model between weather conditions and the *I_c_* index. Our results show that surface pressure and relative humidity can largely predict COVID-19 outbreaks during periods of relatively minor mobility and meeting restrictions. The occurrence of low-pressure systems, associated with the autumn onset, leads to weather and behavioral changes that intensify the virus transmission. These findings suggest that surface pressure and relative humidity are key environmental factors that may be used to forecast the spread of SARS-CoV-2.

## 1 Introduction

Soon after the onset of the COVID-19 disease in December 2019 in Wuhan (China), it became clear that the newly identified causative agent (SARS-CoV-2) had higher transmissivity than the previous coronavirus SARS-CoV-1 and MERSV (1). The declaration of a global pandemic on 11 March 2020 confirmed the worst fears of the high infectiousness capacity of SARS-CoV-2. In this situation, and according to the World Health Organization (WHO), governments implemented a package of measures to deal with the virus. However, by late August 2023 the pandemic of COVID-19 had claimed almost 7 million notified deaths and about 770 million confirmed cases (94), besides the important socio-economic disruptions due to the lockdowns and contention measures. During that time, the scientific community stepped in to help searching for the origins of SARS-CoV-2 and determining the mechanisms that caused its spreading.

As an air-borne disease and with the purpose of developing climate-based predictive tools, the pandemic research soon focused on the climatic and environmental factors that might affect its transmissibility and virulence (2). Several studies explored the relationship between weather conditions and virus transmission, using meteorological or environmental variables as prognostic tools to reproduce the propagation of COVID-19. The different types of models used to predict the spread of the virus are listed in Table 1 along with their associations with the weather variables. Non-linear models include polynomial regressions (PR) and generalized additive models (GAM), incorporating time-lagged weather effects (DLNM). Other efforts have used more simple linear models that work reasonably well, such as single or multiple linear regression (MLR) models and generalized linear (GLM) models. The results show the existence of significant correlations for temperature and relative humidity with virus transmission, with the transmission of SARS-CoV-2 and severity of COVID-19 increasing during dry and cold weather conditions in European countries (Table 1), modulated by large scale atmospheric patterns (3–5). However, the results remain unclear for other variables, such as precipitation, wind speed, solar radiation or surface pressure (Table 1).

**Table 1:** Associations between different weather variables and virus propagation indicated by colors: red (positive), blue (negative), yellow (no association), green (unclear), pink (depending on the value) and grey (not analyzed).

Related Work	Model approach	Spatial Resolution	Temporal Resolution	*T*_mean_	*RH*	*Rad*	*P*	*Prec*	*w*	*T*_min_	*T*_max_
Sanchez-Lorenzo et al. (40)	Non-Linear (PR)	Regional	Monthly								
Fang et al. (73)	Non-Linear (PR)	Regional	Daily								
Gao et al. (74)	Non-Linear (PR)	Global	Daily								
Prata et al. (75)	Non-Linear (GAM)	Regional	Daily								
Qi et al. (76)	Non-Linear (GAM)	Regional	Daily								
Karim and Akter (77)	Non-Linear (GAM)	Regional	Daily								
Fu et al. (3)	Non-Linear (DLNM)	Regional	Daily								
Yuan et al. (78)	Non-linear (GAM)	Global	Daily								
Metelmann et al. (79)	Non-linear (GAM)	Regional	Daily								
Islam et al. (59)	Non-linear (DLNM)	Regional	Daily								
Fong and Smith (80)	Non-linear (DLNM)	Regional	Daily								
Ai et al. (81)	Non-linear (DLNM)	Global	Daily								
Ladha et al. (82)	Linear (MLR)	Local	Daily								
Hu et al. (83)	Linear (MLR)	Regional	Daily								
Hoogeveen et al. (84)	Linear (MLR)	Regional	Daily								
Culqui et al. (4)	Linear (MLR)	Global	Daily								
Matheew et al. (85)	Linear (MLR)	Regional	Daily								
Alnaser et al. (86)	Linear (MLR)	Global	Daily								
Aidaoo et al. (87)	Linear (MLR)	Regional	Daily								
Lin et al. (88)	Linear (MLR)	Global	Monthly								
Liu et al. (89)	Linear (GLM)	Regional	Daily								
Culqui et al. (4)	Linear (GLM)	Regional	Daily								

*Tmean, temperature; RH, relative humidity; Rad, solar radiation; P, surface pressure; Prec, precipitation; w, wind speed; Tmin, minimum temperature; Tmax, maximum temperature.*

It is now clear that external factors such as social behavior (e.g., mobility, use of indoor spaces) and mitigation policies (e.g., use of face masks, vaccination) play the main role in the transmission of SARS-CoV-2 (6, 7). Nevertheless, it has been observed that a small but yet significant fraction of the occurrence and persistence of SARS-CoV-2 is explained by the weather conditions (1–10, 93). When considering the atmospheric variables individually, their predictive skill does not exceed 10%, a figure that increases to about 18% as several variables are considered jointly (8). The complex non-linear interrelation between the weather variables can substantially deviate the estimates obtained from the models. The connection between weather factors is not easily established from observational data, remaining as a challenging task (11). In this context, MLR models are applied to datasets to visualize trends but they have to be analyzed carefully in order to obtain adequate results.

From a mid-term preventive perspective, it is important to improve our capacity to predict seasonal patterns and future outbreaks of SARS-CoV-2, including those new emerging variants with high transmissibility patterns (i.e., Omicron and derivatives). From a long-term adaptative perspective, in the context of the current climate change scenario, a better understanding of the impact of climatic and environmental factors in respiratory diseases becomes crucial to design effective adaptation and mitigation strategies.

The ongoing evolution of SARS-CoV-2 has entered a new scenario where humans are experiencing reinfections with sub-variants of the Omicron lineage, characterized by very high infectiousness. The impact of vaccines on transmissibility of SARS-CoV-2 Omicron variants remains low, as the combination of relatively short immunity against infection and high pathogen genomic variability increase the rate of repeated infection (12, 13) and extend the persistence of coronavirus over time (14, 15). This new scenario has evolved toward an endemic disease, possibly controlled by weather conditions that cause outbreaks or seasonal peaks similar to most common respiratory infections (16, 17).

In the new unfolding epidemiological scenario, where policy interventions and social distancing are already residual, the seasonality of COVID-19 deserves further attention. The predominance of Omicron-related variants, whose infectiousness is highly independent of the vaccinated status, emphasizes the need for a better understanding and characterization of the climatic factors that impact the susceptibility to infection. The dry and warm future scenario for the Euro-Mediterranean region (18) further stresses the need for identifying the main meteorological and environmental precursors for respiratory diseases, and establishing what are their lead mean times.

Here we assess the relationship between the spread of SARS-CoV-2 and the atmospheric conditions in Catalonia (northwestern Mediterranean) from September 2020 to December 2020. A multiple linear regression, based on cross-correlation results between a simple infection index and time-lagged atmospheric variables, is used to model and forecast the impact of weather on the COVID-19 incidence. The model is validated externally during the third COVID-19 outbreak, from December 2020 to February 2021, in order to assess its predictive performance. These results are critically compared with updated findings, on the capacity of environmental factors to predict the seasonal dynamics of the SARS-CoV-2 spread (Table 1).

## 2 Materials and methods

### 2.1 Health data

The health data used in this study are available from the database of the Catalan government (see Data Availability section) and were processed using the MATLAB software (MATLAB R21017a). Due to the limited accuracy of antigen tests, which depends on the time-window considered and results in false negatives, only positive cases detected through PCR (*N_PCR,+_*) were selected for the analysis. Detected cases were grouped into Health Regions (HRs, a total of nine with mean area about 3,600 km^2^) and, for each HR dataset, were broken down into Basic Health Areas (BHAs, a total of 372 with mean area about 86 km^2^ and population ranging between 5,000 and 25,000 people each). Afterwards, a rectangular grid of 0.1° × 0.1° latitude (*lat*) – longitude (*lon*) was generated covering all Catalonia. Health data were assigned to each grid point according to their location in the BHAs, so that grid points located inside one same BHA contain the same health data. BHAs that did not report data during the pandemic were excluded from the preliminary data analysis. As a result, time series for health data at each grid point were generated for the period of time analyzed. These time series were normalized dividing by the area and the population size of each BHA; the resulting series *N_PCR,+_* (*t*, *lat*, *lon*) are positive PCR cases for 100,000 inhabitants and square kilometer (cases per 10^5^ inhab km^2^).

We applied two corrections to the normalized time series *N_PCR,+_* (*t*, *lat*, *lon*). The first one considered the observed weekday biases, e.g., typically as a result of increased counts after the weekend. For this purpose, a histogram of mean confirmed cases for each day of the week at each BHA was computed, and a weekday factor was applied to the previous normalized dataset. A second PCR correction factor was defined as in Equation 1:


(1) $$f_{PCR}=\frac{PCR_{+}\left(t,lat,lon\right)}{PCR_{\mathrm{tot}}\left(t,lat,lon\right)}\text{,}$$

which represents the fraction between the daily positive tests (*PCR_+_*) and the total PCR tests (*PCR_tot_*) done during that day in each BHA region. This factor takes into account the availability of PCR tests, of particular importance during the first wave of the pandemic, as the number of available tests was significantly lower than during the other waves and the number of infected people at the early stages of the pandemic was underestimated.

The normalized time series at each grid point, obtained after applying the two correction factors, were smoothed with a three-day moving average. Further, an interpolant for scattered grid points was applied to estimate daily values in BHAs with no reported cases. The result was a daily COVID-19 health time series ${\hat{N}}_{PCR,+}$ (*t*, *lat*, *lon*) at each point of the grid covering Catalonia.

### 2.2 Daily infection index

The final dataset ${\hat{N}}_{PCR,+}$ (*t*, *lat*, *lon*) was used to define an infection index that monitors the contagion risk for the population in a specific area and day. The daily infection index at each grid point *I_c_* (*t*, *lat*, *lon*) is computed as in Equation 2:


(2) $$I_{c}\left(t,lat,lon\right)=\frac{{\hat{N}}_{PCR,+}\left(t,lat,lon\right)}{\sum_{i=1}^{10}{\hat{N}}_{PCR,+}\left(t-i,lat,lon\right)}\text{,}$$

This pandemic parameter can be obtained directly from the health dataset, providing information on the people infected daily with respect to the total population that is potentially infectious, which is estimated as the people infected during the prior 10 days.

**Figure 1:**
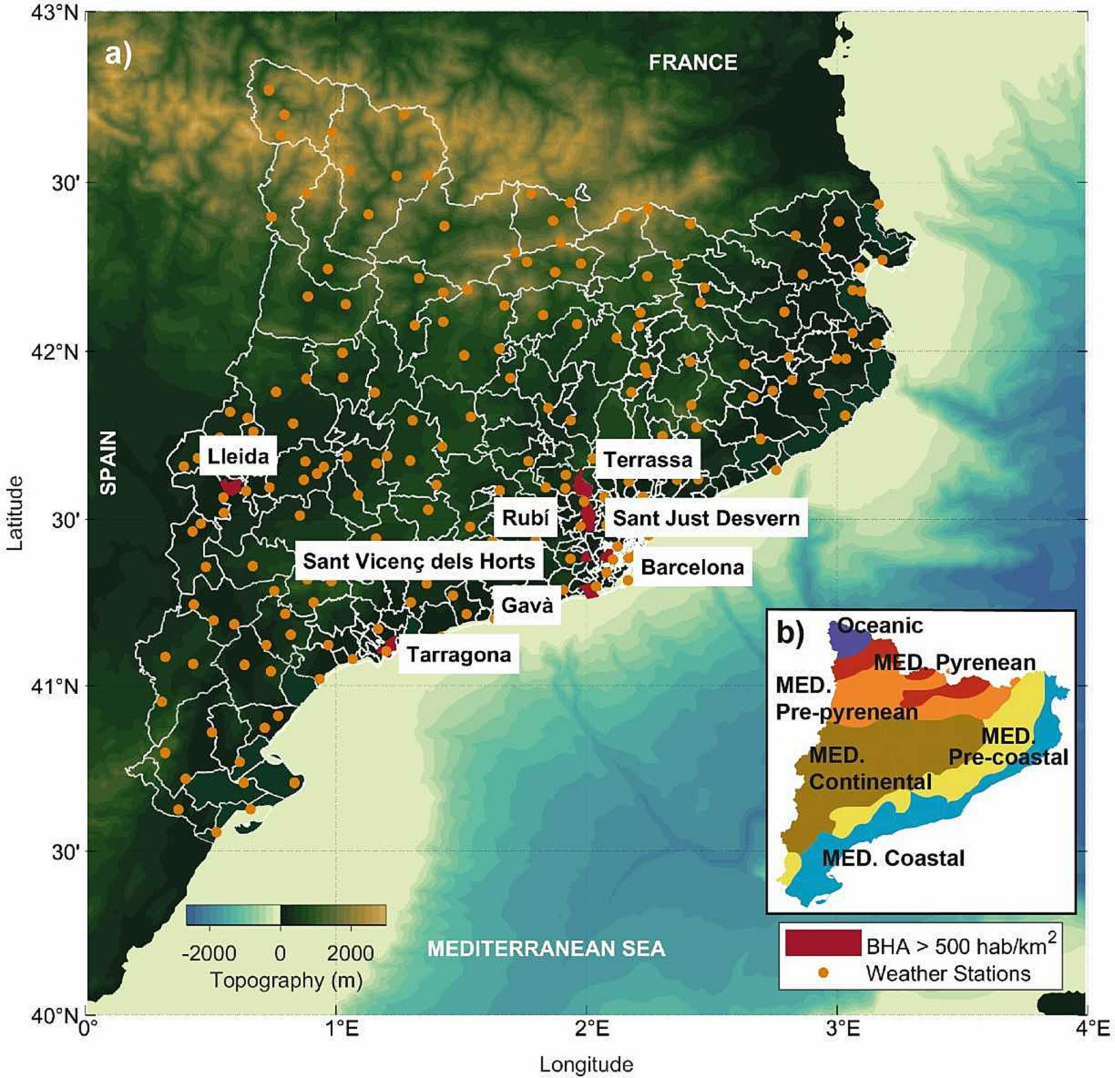
(a) Basic health areas (BHAs, delimited in white) and automatic weather stations (orange dots) in Catalonia. Those BHAs selected for this study, with a population density *d* ≥ 500 inhab km^2^, are drawn in red. The colour bar shows the topography. (b) The inset shows the bioclimates in Catalonia: Mediterranean coastal, Mediterranean pre-coastal, Mediterranean continental, Mediterranean pre-Pyrenean, following the Mediterranean Pyrenean and Oceanic, Meteorological Service of Catalonia (101).

After processing the health data, the BHAs were classified according to population density. In low-density populated areas, during 2020 there were some difficulties in the reporting of positive cases, mostly related to the local availability of tests. In order to avoid this problem, we selected eight densely populated areas (population density *d* ≥ 500 inhab km^−2^) for further analysis (Figure 1). One of the selected BHAs is located in the city of Barcelona (BCN-10A, *d_BCN_* = 11,873 inhab km^−2^) and five more are included in districts located in towns of the Barcelona metropolitan area: Gava (GVA-2, *d_GVA_* = 1,847 inhab km^−2^), Sant Just Desvern (SJD, *d_SJD_* = 2,340 inhab km^−2^), Sant Vicenç dels Horts (SVH-2, *d_SVH_* = 2,409 inhab km^−2^), Rubi (RUB-3, *d_RUB_* = 644 inhab km^−2^) and Terrassa (TRS-E, *d_TRS_* = 1,864 inhab km^−2^). The last two BHAs belong to urban areas away from the city of Barcelona: a district of the town of Tarragona, located by the coast (TRG-2, *d_TRG_* = 1,573 inhab km^−2^), and a district of the town of Lleida, located in the interior of Catalonia (LLEI-2, *d_LLEI_* = 1,123 inhab km^−2^).

### 2.3 Weather data

*In situ* temperature, relative humidity, surface pressure, solar radiation and precipitation data were obtained from a network of 187 automatic weather stations spread along Catalonia and managed by the Meteorological Service of Catalonia (MSC), which is a public institution dependent on the Government of Catalonia (see Data Availability section). For each atmospheric variable, the original 30-min data available since 2009 were averaged to daily values. This allowed estimating the maximum and minimum daily temperatures, the daily thermal amplitude and the difference in mean temperature between consecutive days. The final time series were first smoothed with a 3-day running-average filter and then used to obtain the time series at each BHA by spatial interpolation in the region. In summary, the nine atmospheric variables chosen for assessing the weather impact on the COVID-19 propagation are daily mean values of mean temperature (*T_mean_*), relative humidity (*RH*), shortwave solar radiation (*Rad*), and surface pressure (*P*), as well as daily precipitation (*Prec*), daily minimum (*T_min_*) and maximum temperature (*T_max_*), daily thermal amplitude (*DTA*), and difference in mean temperature between consecutive days (*ΔT*).

### 2.4 Cross-correlation analysis

In June 21, 2020, following a substantial decrease in the number of infections and deaths by COVID-19 and coinciding with the end of the Spanish school year, the Spanish government opened a period with no restrictions in mobility and distancing that was named ‘new-normality’. This allowed a substantial fraction of the Catalan population to spend a few weeks of July–August in holiday destinations. This implied large internal mobility to locations away from their registered residence, disabling a proper normalization of infections in terms of resident population. Hence, we assess the impact of weather on the propagation of COVID-19 immediately after the holiday season, between September 2020 and February 2021. Concretely, an internal validation is done from September 1 to November 18, 2020 – the setup period – which covers a period of relative normality, when most families were back to their homes for work and the start of the academic course, and before the onset of the second COVID-19 wave. Additionally, an external validation is done from November 19, 2020, to February 2, 2021 – the forecast period – which covers a period after the end of the second COVID-19 wave and before a substantial fraction of the population was vaccinated, and includes the third COVID-19 wave.

The relationship between the local weather and health variables is explored through a time-lagged cross-correlation analysis between each of the nine atmospheric variables and the *I_c_* index. To evaluate the similarity between the two series, the atmospheric variables are shifted backwards in time with respect to *I_c_*. Following our initial hypothesis, that infection is driven by weather conditions, only negative lags are considered for further analysis; these negative time lags (*τ* < 0) indicate that changes in the infection index follow the atmospheric variables. According to the average maximum reported COVID-19 incubation days (91) and to the *I_c_* definition, the maximum time lag selected was *τ_max_* = 10 days. This lead–lag correlation analysis will allow us to determine the lead time of each weather parameter that affects the COVID-19 transmission. In order to quantify the impact of weather on the spread of the virus, we assume that an atmospheric variable affects the virus propagation if the sample correlation coefficient (*CCF*) between this variable and the infection index is significant below a threshold of ɑ = 5%.

### 2.5 Selection of climatic variables to build the model

Once the weather precursors and their characteristic lead time are identified, the COVID-19 propagation is modeled using a MLR model for each BHA. This model expresses the predicted infection index *I_c,pred_* in terms of *p* local climatic predictors [Equation 3]:


(3) $$I_{c,\mathrm{pred}}\left(t;\;X_{1},\ldots ,X_{p}\right)=c_{0}+\sum_{j=1}^{p}c_{j}\cdot X_{j}\left(t+\tau_{j}^{*}\right)\text{,}$$

where *t* is time, *X_j_* indicates any of the local predictors of the model, *c*_0_ is the constant coefficient for the model, *c_j_* (*j* ∈ [1, *p*]) is the regression coefficient for the *X_j_* predictor, and $\tau_{j}^{*}$ is the characteristic lag for the *X_j_* predictor. The characteristic time lag (or lead time) is defined as the time interval for which the highest correlation coefficient between the predictors and the observed $I_{c}$ index is obtained. Hence, a total of 2*p* + 1 parameters are obtained for each BHA.

### 2.6 Building the model: model descriptors and statistics

Before building the model, we explore the potential collinearity effects between the predictors as detailed next. First, the correlation coefficients *r_i,j_* between two predictors, namely *X_i_* and *X_j_*, are computed. Second, correlation *t*-tests are done to evaluate whether the predictors have a significant linear relationship. The *t*-statistic *t_TS,ij_* associated with each combination of predictors is calculated as in Equation 4:


(4) $$t_{TS,ij}=\frac{r_{i,j}\sqrt{n-2}}{\sqrt{1-r_{i,j}^{2}}}\;\text{,}$$

where *n* is the size of the sample. The statistics follows a *t*-distribution with *n* - 2 degrees of freedom *t_TS,ij_* ≅ *t*_*n-*2_. Finally, if the two predictors are correlated, the degree of collinearity between them is evaluated using the variance inflation factor, defined as in Equation 5:


(5) $$VIF_{j}=\frac{1}{1-r_{j}^{2}}\text{,}$$

where the parameter *r_j_* indicates the coefficient of determination of the variable *j* regressed on the remaining predictors. If *VIF_j_* is less than 2.5, we consider that collinearity is not significant and both predictors can be used to build the model (19).

After assessing the collinearity between predictors, we test the regression coefficients separately for each BHA in order to select the weather predictors included in the final model. The significance for the regression coefficients is assessed using the *t*-test. Since these tests can be very conservative, we apply the forward stepwise estimation method (20) to decide whether a candidate predictor must be included in the model, as follows. First, we select the predictor with the highest correlation coefficient with the infection index, and fit this predictor and the infection index to a linear regression. As the model is linear, we use the adjusted coefficient of determination, *r*^2^*_adj_*, to evaluate the goodness of the fit. In our case, this coefficient represents the percentage of the variation in the infection index that can be explained by the variation in the predictors, taking into account the size and the number of independent variables in the model. Next, the predictor that has the second highest correlation with the infection index is included in the linear model and *r*^2^*_adj_* is recomputed. The partial *F*-test statistics is used to decide whether the addition of that predictor makes a significant contribution to the model, calculated as in Equation 6:


(6) $$F_{k_{2}-k_{1},n-k_{2}-1}=\frac{\frac{\left(SSR_{2}-SSR_{1}\right)}{\left(k_{2}-k_{1}\right)}}{\frac{SSE_{2}}{\left(n-k_{2}-1\right)}}\text{,}$$

where the sub-indexes 1 and 2 correspond to the models with the remaining predictor removed or added, respectively. The terms *SSR* and *SSE* indicate the sum of the squares due to regression (i.e., the variability of *I_c_* explained by the regression) and the sum of the squares due to error (i.e., the variability of *I_c_* not explained by the regression) for the corresponding models. Finally, *n* represents the size of sample and *k* the amount of predictors used in the corresponding linear regression. The *F*-statistic is inspected at ɑ = 10%. If the *F*-value is significant at this level, the model improves significantly with the addition of the new predictor, which is hence maintained in the model. This second step of the forward stepwise (FS) method is repeated for the remaining predictors, which are added one by one from higher to lower correlation. The final model includes all predictors that pass this partial *F*-test.

The FS method has been widely used to select the set of variables to be included in the models because it is easy to implement and reduces the model complexity removing irrelevant or redundant variables (21–23). However, this method has been also questioned to have an inherent bias (24, 25, 95, 96) because it assigns the variables with the highest correlations a higher chance to end up in the final model (26, 27). After considering the potential drawbacks of the method, our model has been assessed by means of a new model constructed using the unbiased two-step method (24). The results obtained from this linear model at each specific BHA are compared to the results of the FS model (see Supplementary Information) showing similar results.

The final FS model is assessed using the joint *F*-test. This test allows deciding whether the linear regression used in the model provides a better fit to the observations than a model with no predictors (intercept-only model). The test statistic, which is denoted by *F*, has a Fisher distribution that is calculated as in Equation 7:


(7) $$F=\frac{SSR}{SSE}\frac{\left(n-k-1\right)}{k}$$

where *SSR*, *SSE* and *n* are defined in Equation 6 and *k* is the number of predictors in the model. For each BHA, the linear fit to the data allows obtaining the *F*-statistic along with its corresponding *p* value. If the *p* value is higher than a 5% significance level (ɑ), we conclude that the final model fits the observations better than the intercept-only model.

Finally, the assumptions in the linear regression model are analyzed through the residuals from the fitted model; statistical tests are implemented to complement the graphical information. First, the Kolmogorov–Smirnov (K-S) test is conducted at ɑ = 5% in order to examine if the residuals are normally distributed (28). This allows assessing whether the gap between the empirical and normal (hypothesized) distributions of the residuals is significant at the considered significance level. In addition, the White test for heteroscedasticity (29), with ɑ = 5%, is used to assess if the regression errors have a non-constant variance. If the *p* value associated with the test statistic, which follows a Chi-square *χ*^2^ distribution, is smaller than the significance level of the test, then there is no evidence of heteroscedasticity in the final model.

### 2.7 Model validation during the setup period

The final model is internally validated for the setup period, from September 1 to November 18, 2020, through the implementation of the leave-one-out cross-validation (LOOCV) method. In the LOOCV procedure, the infection index *I_c_* for the day *i* (*I_c,i_*) is excluded and the model fitted using the remaining data. Each of these training subsets provides an individual model, which is expected to be slightly different from the original one, and then these models are used to predict the infection index *I*_*c,*[*i*]_ with the *i*-th case removed from the sample. The prediction error from these models, computed as *e*_[*i*]_ = *I_c,i_* - *I*_*c,*[*i*]_, is a measure of how close the prediction is to the observation when this observation is omitted. The absolute percentage error in each measurement is obtained scaling each prediction error against its corresponding observed value: *APE*_[*i*]_ = 100|*I_c,i_* – *I*_*c,*[*i*]_|/*I*_*c,*[*i*]_.

The overall performance of the model is estimated using the mean of the absolute percentages previously calculated (*MAPE_CV_*). Then, the value of that mean (*MAPE*_CV_) is compared to the mean of the absolute percentages obtained when the model was built (*MAPE*) in order to internally evaluate the consistency of the model. Finally, other statistical parameters are determined from the linear regression fit of the observed values *I_c,i_* to the cross-validated ones *I*_*c,*[*i*]_: a significance test assesses the deviations of the slope and the *y*-intercept to the expected values, which are *β*_1_ = 1 for the slope and *β*_0_ = 0 for the *y*-intercept, respectively; a linear fit allows computing the R-squared of the cross-validation, *q*^2^*_CV_*; and an *F*-test of overall significance tells whether the predictions explain a significant part of the variance observed in the responses as compared to data obtained from a model with no predictors.

The statistical parameters obtained by the application of the LOOCV method are finally compared to the corresponding ones obtained from the data used to build the model. Table 2 summarizes the expressions of the statistical parameters used for construction and validation of the model (30).

**Table 2:** Summary of the expressions used to determine the statistic parameters (*MAPE*, *RMSE*, standard R-squared *r*^2^ and its adjusted version *r*^2^*_adj_*) in the internal and external model validations (left), and the internal cross-validation using the LOOCV method (right).

Model building and External validation	LOOCV (Internal validation)
$\mathrm{MAPE}\;=\;\frac{1}{n}\sum_{i=1}^{n}APE_{i}=\;\frac{100}{n}\cdot \sum_{i=1}^{n}\;\frac{\left|I_{c,i}\;-I_{c,\mathrm{pred},i}\right|}{I_{c,i}}$	$\mathrm{MAP}E_{CV}=\frac{1}{n}\sum_{i=1}^{n}\;APE_{\left[i\right]}=\frac{100}{n}\cdot \;\sum_{i=1}^{n}\;\frac{\left|I_{c,i}\;-\;I_{c,\;\left[i\right]}\;\right|}{I_{c,i}}$
$\mathrm{RMSE}\;=\;\sqrt{\frac{\sum_{i=1}^{n}\;e_{i}^{2}}{n}}=\sqrt{\frac{\sum_{i=1}^{n}\;{\left(I_{c,i}\;-\;I_{c,\mathrm{pred},i}\right)}^{2}\;\;}{n}}$	$RMSE_{CV}=\;\sqrt{\frac{\sum_{i=1}^{n}\;e_{\left[i\right]}^{2}}{n}}=\;\sqrt{\frac{\sum_{i=1}^{n}\;{\left(I_{c,i}\;-\;I_{c,\;\left[i\right]}\right)}^{2}\;\;}{n}}$
$r^{2}=\;1\;-\;\frac{\sum_{i=1}^{n}\;{\left(I_{c,i}\;-\;I_{c,\mathrm{pred},i}\right)}^{2}\;\;}{\sum_{i=1}^{n}\;{\left(I_{c,i}\;-\;I_{c}\right)}^{2}}$ $r_{adj}^{2}\;=\;1-\left(1-r^{2}\right)\;\frac{n-1}{n-k-1}$	$q_{CV}^{2}\;=\;1\;-\;\frac{\sum_{i=1}^{n}\;{\left(I_{c,i}\;-\;I_{c,\;\left[i\right]}\right)}^{2}\;\;}{\sum_{i=1}^{n}\;{\left(I_{c,i}\;-\;I_{c}\right)}^{2}}$

### 2.8 Model validation during the forecast period

The final model is generalized using independent meteorological and health datasets for the period from the end of the setup period until February 2021. This forecast period, which includes the pandemic’s third wave in Catalonia, allows testing the predictive skill of the model in each of the BHAs. Mobility and social restrictions in Catalonia were reduced at the end of November 2020, followed by an increase of positive cases (third wave) that led to some restrictions on mobility (including curfew at night) and restaurants. The number of positive cases peaked about January 15, 2021, followed by a decrease in the number of infections, in February falling to those levels previous to the onset of the third wave. On the other hand, vaccination in Catalonia began on December 27, 2020. The percentage of people who had received their first vaccine remained low (< 10%) until February, and increased to about 30% by the end of April 2021. Despite this minor fraction of the vaccinated population and the reset of some mobility limitations, we have considered the data from November 19, 2020, to February 2, 2021, to validate the final model for all eight BHAs.

The validation for this forecast period is done following the same procedure as described for the internal validation. First, the values of the infection index *I_c,i_* during this time period are calculated from the health data time series. Second, the predicted values *I_c,pred,i_* are estimated from the multiple linear regression model. Third, the predicted and observed values are compared and the statistical significance of the linear fit, the slope and the intercept are assessed. Finally, the prediction errors for each value of *I_c,i_* are computed as the differences between observed and predicted values, *e_i_* = *I_c,i_* - *I_c,pred,i_*, normalized by each observation *I_c,i_* in order to obtain the mean absolute percentage error of the external validation (*MAPE*). Additionally, the same statistical parameters estimated for the internal validation are calculated to assess the overall performance of the final model with this new dataset, including *r*^2^*_ext_*, *r*^2^*_ext,adj_* and *RMSE* (Table 2).

## 3 Results

### 3.1 Seasonality of COVID-19 in Catalonia

**Figure 2:**
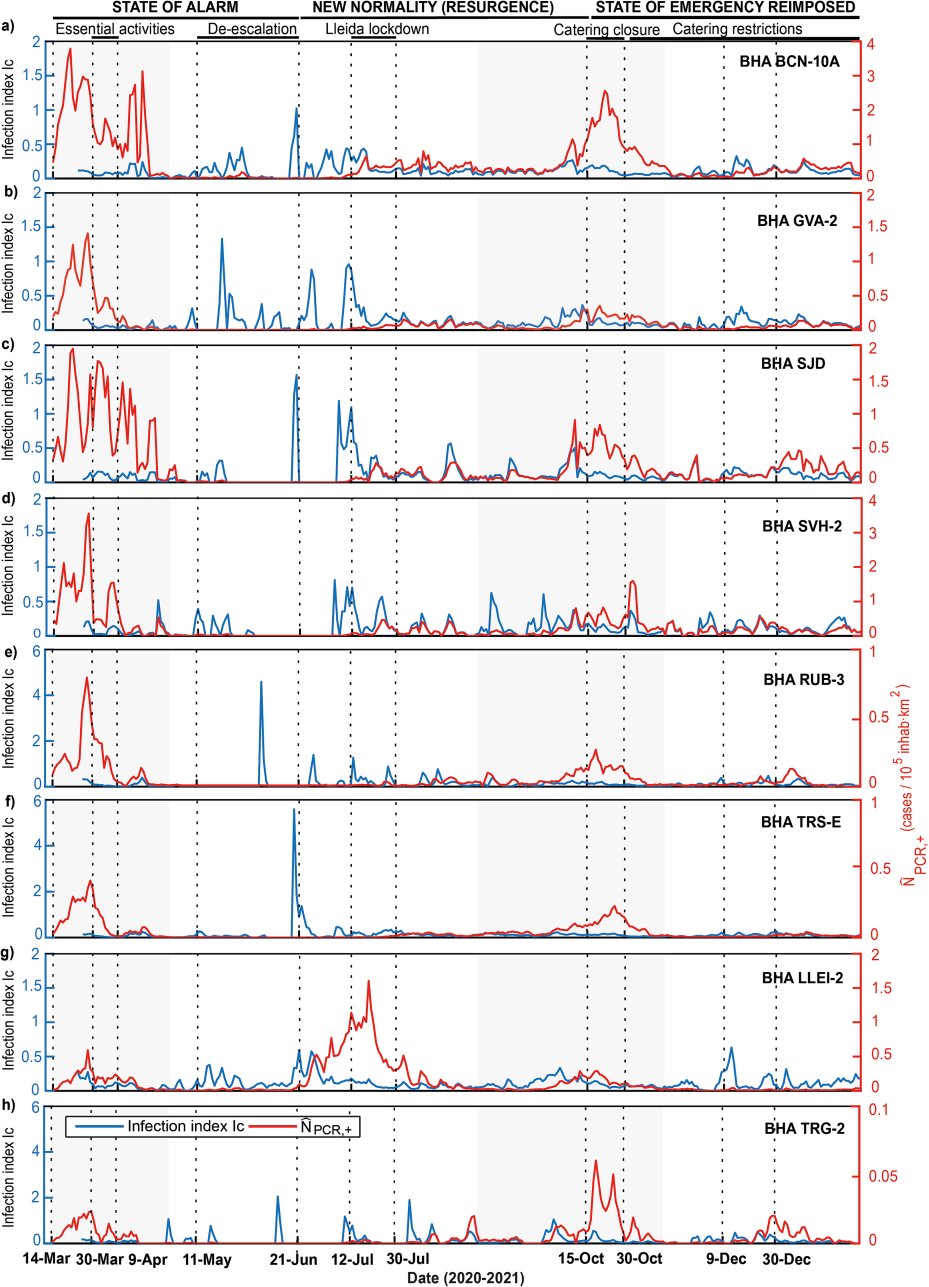
(a-h) Temporal evolution during 2020 and beginning of 2021 of the number of COVID-19 cases in the eight selected Catalan BHAs, presented both in terms of the normalized number of cases ${\hat{N}}_{PCR,+}$ as determined through the PCR positive tests (red line) and the rate of infections *I_c_* (blue line). In each panel, the gray shaded areas indicate periods that include the first and the second pandemic waves and the vertical dotted lines delineate the duration of the main social and mobility restrictions imposed in Catalonia.

The first and second COVID-19 outbreaks in Catalonia took place, respectively, in March–April and October 2020. Both outbreaks have been assessed through the number of positive PCR cases, normalized in terms of population and area, and the infection *I_c_* index (see Methods) (Figure 2). Values of *I_c_* higher than 1 indicate that the number of positive cases at any day were higher than the summation of all positive cases during the previous 10 days, characterizing situations of high transmissibility for small number of infections. Note that the range of normalized PCR cases varied substantially between locations, from maximum values exceeding 3.5 per 10^5^ inhab km^2^ for the most populated values (Figures 2a,d) and decreasing to peak values about 0.05 cases per 10^5^ inhab km^2^ in the least populated BHAs (Figure 2h). However, the peak infection index ranged between about 1 and 6, with larger and more intermittent peaks in the least populated BHAs.

The Spanish first state of alarm lasted between March 14 and June 21, 2020, with strict social interaction and mobility restrictions. After this last date and until the end of October, these measures were similar to the pre-pandemic period, leveraged by non-pharmaceutical interventions (NPI) like minor mobility restrictions and compulsory face masks. The Spanish holiday season takes place in July and August, characterized by major mobility from high- to low-density rural and coastal areas, a fact that affects substantially the demography and hardens any normalization effort. For this reason, for our analyses we have focused on two post-holiday periods, as discussed below.

During the first pandemic wave, the BHA in Barcelona (BCN-10A) reached the highest number of confirmed cases with 3.9 cases per 10^5^ inhab km^2^ followed by the BHAs of Sant Vicenç dels Horts (SVH-2) and Sant Just Desvern (SJD) with 3.7 and 1.9 cases per 10^5^ inhab km^2^, respectively (Figures 2a,c,d). These maxima occurred before the Spanish government banned all non-essential activities on March 14. Following this first lockdown and coinciding with the lowest percentage of mobility registered during 2020 (31), the number of confirmed cases sharply decreased in all BHAs. During the first half of April the number of positive cases diminished progressively for Gava (GVA-2), Rubi (RUB-3), Terrassa (TRS-E) and Lleida (LLEI-2) (Figures 2b,e,f,g), whereas for the remaining BHAs (with the highest density population) there were still several intermittent important peaks (e.g., BCN-10-A and SJD, Figures 2a,c). In the second half of April the number of cases reduced drastically, flattening the curve for all BHAs and leading to a gradual leverage of social restrictions and an increase in mobility. In June 2020, when most of the mobility restrictions had stopped, the number of positive PCR remained low, not exceeding 0.5 cases per 10^5^ inhab km^2^. The single exception was LLEI-2, which reached 1.5 cases per 10^5^ inhab km^2^ (Figure 2g). In this particular case, the enhancement in virus transmission was associated with seasonal agricultural workers living in overcrowded conditions, which acted as reservoirs and further spreaders of the infection (32).

During this first wave the *I_c_* index remained low, indicating that the government measures were effective to prevent contagion. In contrast, several high *I_c_* peaks appeared intermittently during summer (Figure 2) without any major response on the standardized PCR, with the exception of LLEI-2 (Figure 2g). This suggests that, despite the existence of several irregular infection episodes, the initial low numbers of infected people and the intermittency of these events did not allow the number of infected people to grow. It could be argued that the weather conditions were neither favorable to spread the infection but, because of the extremely high mobility during this period, this is very difficult to assess.

During the second pandemic wave, the highly-populated Barcelona metropolitan area (BCN-10A, SJD and SVH-2) showed again the highest values, respectively with 1.7 and 2.7 cases per 10^5^ inhab km^2^, although these values were lower than during the first wave of the pandemic (Figures 2a,d). In contrast, in the coastal town of Tarragona (TRG-2) values reached 0.07 per 10^5^ inhab km^2^, higher than during the first wave (Figure 2h). The normalized number of positive PCR cases behaved similarly in all BHAs, rising in the second half of September and peaking in late October. Throughout summer, the *I_c_* index remained intermittent and relatively high in all BHAs except Terrassa (TRS-E) and Rubi (RUB-3). The mobility restrictions remained low until the end of October, suggesting that the increase in COVID-19 transmission could have been influenced by weather conditions (33, 34). Indeed, during this time period, several cold fronts circulated from west to east in a row, a typical autumn scenario (Supplementary Figures S1, S2).

### 3.2 Correlation between weather variables and the infection index

In order to explore the role of weather conditions on the second COVID-19 wave in our study region, we analyze the time-lagged correlations between local infection indicators and weather data for each of the eight BHAs. A total of nine daily-averaged atmospheric variables for each BHA are used during the period from September 1 to November 18, 2020 (see Methods). The initial selection of humidity and temperature is based on previous research on SARS-CoV-2 and other respiratory viruses such as influenza, which explored the impact of seasonal variations of these variables on virus survival in the environment or on host susceptibility (35, 36, 97). Additionally, we include daily mean values of solar radiation, and surface pressure, as well as daily precipitation, daily minimum and maximum temperature, daily thermal amplitude and the difference in mean temperature between consecutive days.

**Figure 3:**
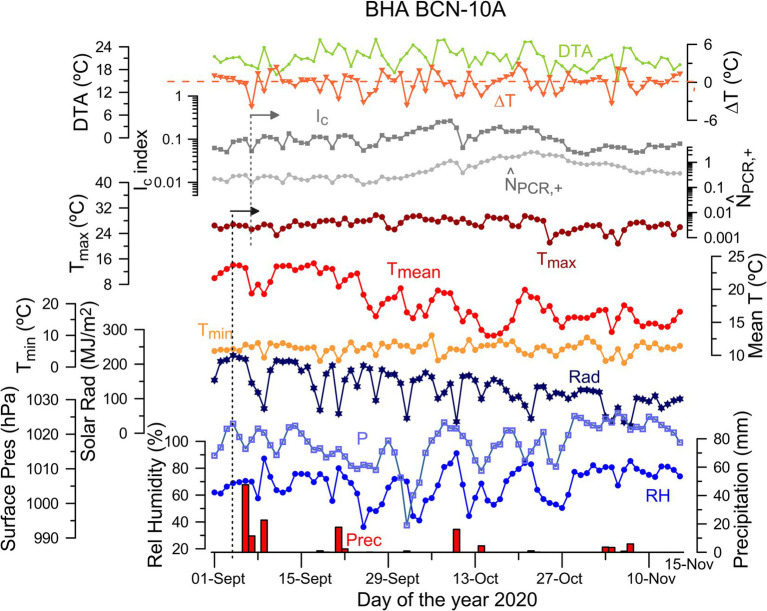
Time series of daily values of atmospheric variables during the period that includes the second outbreak (September 1 to November 18, 2020) together with the normalized number of cases ( ${\hat{N}}_{PCR,+}$ ) and the infection index (*I_c_*) for BCN-10A. The atmospheric daily variables are mean temperature (*T_mean_*), relative humidity (*RH*), solar radiation (*Rad*), precipitation (*Prec*), surface pressure (*P*), minimum and maximum temperature (*T_min_*, *T_max_*), daily thermal amplitude (*DTA*) as well as the difference in mean temperature between consecutive days (Δ*T*). The units for these variables are indicated in their corresponding axes. The vertical black and gray dashed lines indicate the arrival of a cold front on September 7 and the changes observed in the health series a few days later.

The evolution of the normalized number of cases and the infection index is compared with changes in the atmospheric variables, as shown in Figure 3 for BCN-10A (see Supplementary Figure S2, for all BHAs). Oscillations in surface pressure, temperature, relative humidity and solar radiation are associated with the passage of cold fronts in this area (e.g., the September 7–10 variation in Figure 3). The time series also reveals changes in the infection index, which seem to show up several days after the atmospheric changes (gray dashed line in Figure 3). We hence explore the correlation of *I_c_* with the entire selected set of atmospheric variables in order to determine their possible influence on the spread of the virus.

Our results show consistent significant negative correlation of surface pressure (*P*) and relative humidity (*RH*) with the lagged infection index *I_c_* for all BHAs. A negative correlation indicates that a decrease in *P* and/or *RH* enhances the spread of the virus several days later. For *P* – *I_c_*, the correlation coefficients are statistically significant (*p* < 0.01) in all BHAs for specific time lags (*τ_P_*). The cross-correlation *P* – *I_c_* coefficients are high for all BHAs (*CCF* ≅ − 0.5), with a time lag fairly constant at about – 7 days, although some areas have even higher coefficients at shorter lags (Supplementary Figure S3). For *RH* – *I_c,_* the cross-correlation coefficients are statistically significant with *p* < 0.01 in four of the BHAs (GVA-2, SJD, SVH-2 and TRS-E) and at *p* < 0.05 in the remaining four BHAs. In this case, the highest values for the correlation coefficients (*CCF* ≅ − 0.5) correspond to *τ_RH_* between −5 to −3 days (Supplementary Figure S3). It means that an increase of the infection index in Catalonia is preceded by low surface pressure (7 days before) and dry conditions (3–5 days before). The other meteorological variables evaluated do not show consistent correlation scores (Supplementary Figure S3). In particular, the variables derived from temperature and precipitation are poorly correlated with the infection index, except for LLEI-2 and TRG-2 that have significant correlations at *τ* = −10 days. The daily thermal amplitude is significant (*p* < 0.05) in five out of the eight BHAs, but the cross-correlation function for these BHAs has variable time lags. A similar situation appears in the case of the shortwave solar radiation (Supplementary Figure S3). These results suggest that daily thermal amplitude and shortwave radiation can contribute additionally in the COVID-19 transmission in specific locations. Hereafter, we focus on the common meteorological patterns that affect and can be used as predictors for our entire area of study.

**Figure 4:**
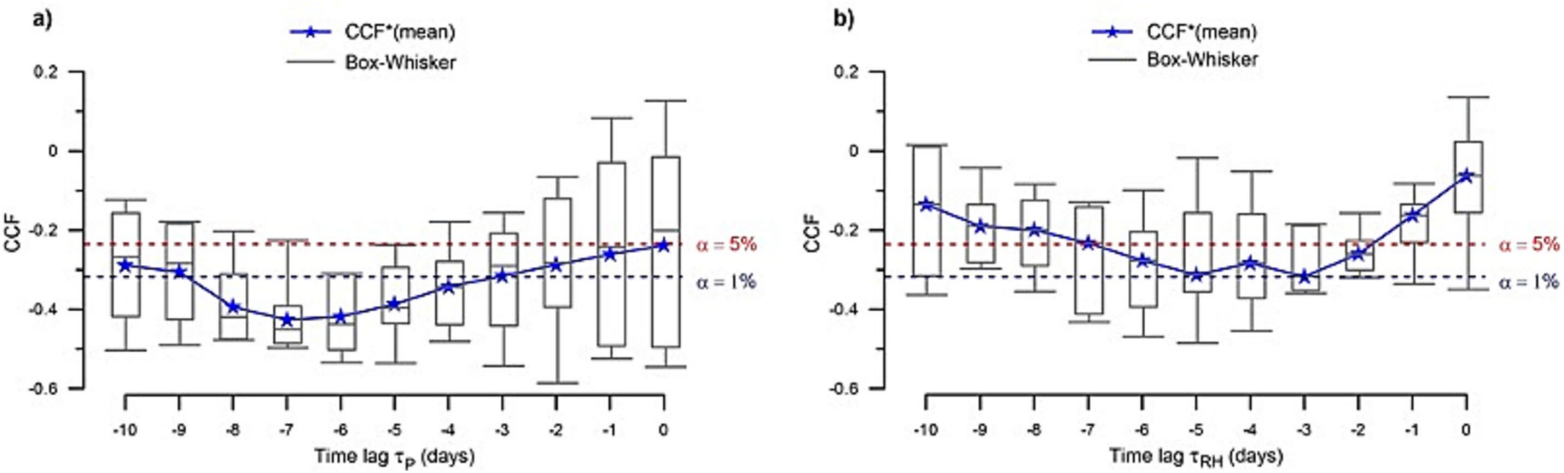
Composite box plots of the cross-correlation coefficients (*CCF*) of (a) surface pressure and (b) relative humidity with respect to *I_c_* as a function of lag time, calculated using the eight reference BHAs. The lower and upper ends of the box represent the first and third quartiles, respectively, and the mean (CCF^*^) is indicated by a blue star. The whiskers extend to the most extreme value within 1.5 IQR (interquartile range) from the box ends. Horizontal dashed lines indicate the statistical significance of the coefficients at ɑ = 5% (red line) and ɑ = 1% (blue line).

We use all eight BHAs to produce a composite box-plot of *CCF* as a function of time lag, for either *P* and *RH* with respect to *I_c_* (Figure 4). For each lag, we select the mean of the *CCF* values for each box plot (hereinafter CCF^*^) as the representative value of the set. For both variables, CCF^*^ shows a well-defined valley where the negative correlation is highest (Figure 4). Surface pressure has the largest absolute correlation CCF^*^ = − 0.42 at *τ_P,min_* = − 7 days (with standard deviation std(*CCF*) = 0.08) and the relative humidity has minima at both *τ_RH,min_* = − 3 (CCF* = − 0.28, std(*CCF*) = 0.08) days and - 5 days (CCF* = − 0.27, std(*CCF*) = 0.14). For both variables, the *τ_min_* values occur at CCF^*^ significant levels.

For surface pressure, *CCF* ^*^ is actually significant at ɑ = 10% for *τ_P_* ∈ [−8, −4]; in particular, the smallest interquartile ranges (IRQ) for the *CCF* distributions are in this lag interval (IRQ ≅ 0.1), indicative of a minimum dispersion of the *CCF* values among the different BHA (Figure 4a). The smallest dispersion is found at *τ_P,min_* = − 7 days, with IRQ (*τ_P,min_*) ≅ 0.05 and std(*CCF*) =0.08. We conclude that most of the inspected BHAs show the highest correlations between $I_{c}$ and P at *τ_P,min_* = − 7 days.

In the case of the relative humidity, we observe a similar behavior, with CCF^*^ values statistically significant in a range of *τ_RH_* ∈ [−6, −2] days (Figure 4b). In this interval, the interquartile range (IQR) for the *CCF* coefficients is about 0.2, and the smallest value takes place at *τ_RH_* ≅ − 3 days, with IRQ (*τ_RH,min_*) ≅ 0.1 (std(*CCF*) = 0.08).

These results indicate that the infection index was negatively correlated with the surface pressure conditions 7 days before and with the relative humidity conditions about 3 days before. We conclude that a decrease (increase) in *P* or *RH* leads to an increase (decrease) in *I_c_* and vice versa some 7 and 3 days later, respectively. These values for the time lags are chosen as the characteristic lags (τ^*^) for the surface pressure and the relative humidity in the study area.

### 3.3 Surface pressure and relative humidity as predictors of COVID-19 variability

The cross-correlation analysis reveals that surface pressure and relative humidity are the only meteorological variables that bear statistical significant correlation with *I_c_* (*p* < 0.05) in all BHAs. Therefore, the model is built using only two candidate predictors (*P* and *RH*), hence requiring five parameters for each BHA (Equation 3). However, the results of the composite plots of the *CCF* (see section 3.2) evidence similar time lags for each predictor over the entire region. The substantial variations in the coefficients suggest that the intensity of the response is region-dependent, likely reflecting specific demographic and geographic characteristics (Figure 1), but the similarity of the time lags advocates for the existence of analogous responses to environmental forcing. Hence, despite the infection indexes differ substantially between different BHAs, we propose a multiple linear regression model of the infection index (Equation 3) in terms of these two variables, with changing coefficients but one same time lag for all BHAs.

This approach reduces the total number of free parameters for each BHA to only three, so the model is expressed as in Equation 8:


(8) $$I_{c,\mathrm{pred}}\left(t;P,RH\right)=c_{0}+c_{1}\cdot P\left(t+\tau_{P}^{*}\right)+c_{2}\cdot RH\left(t+\tau_{RH}^{*}\right)$$

where *c*_i_ (∀i ∈ [0,2]) are the model intercept and regression coefficients for each BHA, the predictor 1 corresponds to the surface pressure *P* (measured in hPa) and the predictor 2 corresponds to relative humidity *RH* (measured as a percentage of absolute humidity relative to the maximum saturation value for that temperature). The temporal variable (*t* ≥ 0) is the day counter for the selected time period and τ^*^_j_ is the characteristic time lag for the *j* predictor. These lags between the weather and health variables indicate the leading times of the atmospheric parameters in the COVID-19 transmission. In practice, it means that the atmospheric time series cover the period from September 1 to November 15, 2020, while the health time series refer to the period September 8 to November 18, 2020.

The setup and application of the climate-dependent COVID-19 model is explained in detail in Methods. Briefly, the model is first developed using the *P*, *RH* and *I_c_* time series for a period that includes the second outbreak (September 1 to November 18, 2020, the setup period), obtaining the time lags and regression coefficients for each BHA. Since the FS method may possibly introduce some bias in the regression coefficients (see section 2.6 in Methods), the results of our model have been compared with those from a simpler unbiased method, where all weather variables are simultaneously assessed. The comparison between the two models shows that the bias introduced in our FS model is negligible.

After this validation, the FS model is used to forecast the infection index *I_c,pred_* for an independent dataset (November 19, 2020, to February 2, 2021, the forecast period). The validations for both the setup and forecast periods are conducted through the leave-one-out cross-validation (LOOCV) method (see External validation of the model in the Supplementary Information). Note that after February 2021, over 10% of the Catalan population had already received their first vaccine (37), undermining the use of more recent data for external validation. The results and statistics of the model validation are extensively explained in the Supplementary Information.

Fitting the model infection index *I_c,pred_* predictions to the observations for the setup period (see Model parameters and statistics in the Supplementary Information) shows that the two predictors, *P* and *RH*, have a significant contribution to the model (*p* < 0.1) in four of the eight BHAs (BCN-10A, SJD, RUB-3 and LLEI-2). For the other four stations, it turns out that one single predictor, *P* or *RH*, is enough to characterize the evolution of *I_c_*. Specifically, the model does not significantly improve by adding *P* in TRG-2 or by adding *RH* in GVA-2, SVH-2 or TRS-E. The regression coefficients, *c*_1_ and *c*_2_, are significant (*p* < 0.05) in all BHAs and their values vary between [−10, −3] × 10^−3^ hPa^−1^ (mean(*c*_1_) = −5.90 × 10^−3^ hPa^−1^; std(*c*_1_) = 1.90 × 10^−3^ hPa^−1^) and [−4, −1] × 10^−3^ (mean(*c*_2_) = −1.91 × 10^−3^; std(*c*_2_) =1.12 × 10^−3^). The negative regression coefficients indicate that a decrease (increase) in the infection index occurs when *P* and *RH* increases (decreases) several days before, confirming the results of the correlation analysis (Figures 4a,b).

During the pandemic second outbreak or setup period, as expected, the model captures moderately well the general behavior of *I_c_*. This is confirmed by the significant correlation coefficients (*r*), derived from the R-squared (*r*^2^) of the corresponding linear regressions between the *I_c,pred_* and *I_c_* (Supplementary Figure S5 and Supplementary Table S19). In particular, RUB-3, TRS-E or BCN-10A exhibit the highest correlations, with *r* = 0.67, 0.54 and 0.56, respectively; SVH-2 and LLEI-2 show correlations higher than 0.5, and the remaining three BHAs show lower yet statistically significant correlations (0.3 < *r* < 0.5; *p* < 0.01). We conclude that our simple climate-dependent model reproduces several main changes in the infection index during the second COVID-19 outbreak in Catalonia (fall 2020).

### 3.4 Forecasting the infection index during the third wave

**Figure 5:**
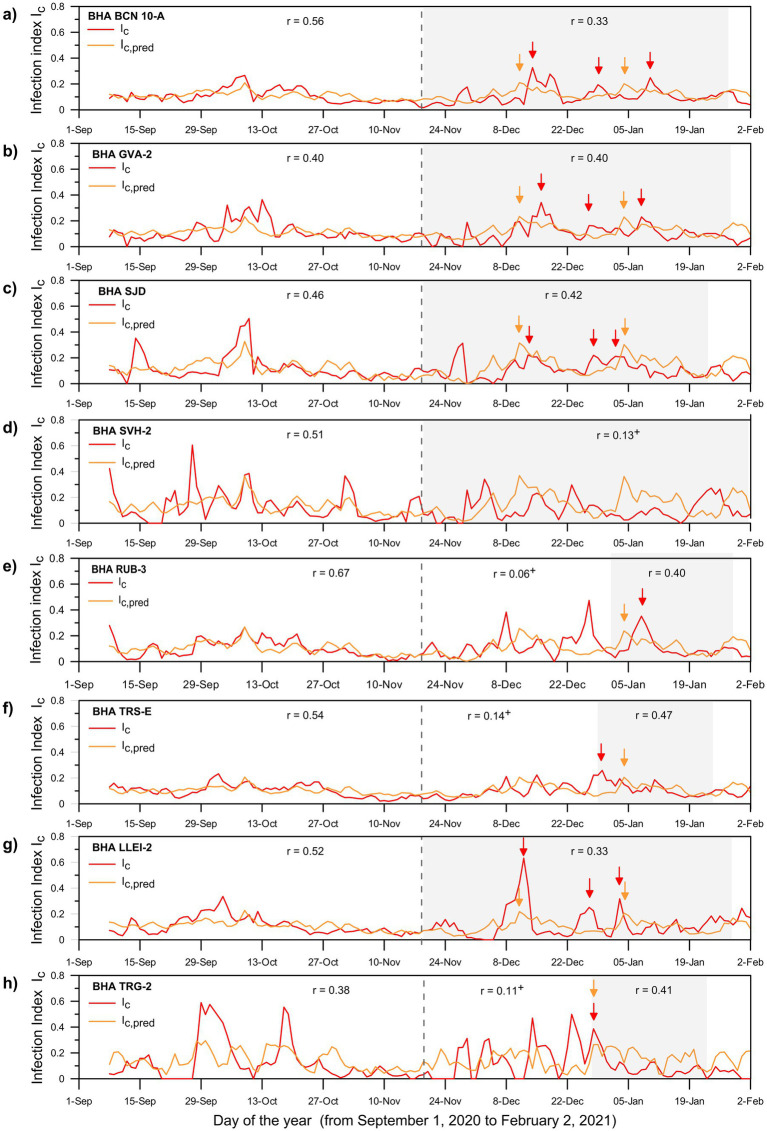
Results for the eight BHAs (panels a through h) showing the temporal evolution of the observed *I_c_* (red lines) and predicted *I_c,pred_* (orange lines) infection index for the period between September 1, 2020, and February 2, 2021. The vertical dashed line on November 18, 2020, separates the setup and forecast periods. The gray shaded areas indicate selected portions of the forecast interval with the highest correlation coefficient between the two series. The correlation coefficients for each time interval are displayed in the upper part of each panel. The symbol ‘^+^’ indicates time intervals when the two series are not statistically significant at a maximum ɑ of 1%; in several cases it corresponds to time periods of low normalized number of cases (Supplementary Figure S6). The arrows indicate the observed (red) and predicted (orange) *I_c_* peaks during the forecast periods.

The pandemic third outbreak started with an increase of the infection rate in early December 2020 but declined to the pre-outbreak levels in January 2021 (red lines, Figure 5). The mobility and social measures progressively relaxed after November 2020 and the state-of-alarm was revoked in early May 2021. Social interactions increased substantially during Spanish public holidays in early December and Christmas time, leading to a third wave that had specific features for each BHA. For example, in BCN-10A, GVA-2 and LLEI-2, the infection rate shows a marked peak in mid-December followed by two secondary peaks in late December and early January (Figures 5a,b,g). However, this is not the case for SJD, which had peaks of comparable amplitude (Figure 5c), or for TRS-E, where the situation was reversed and the two secondary peaks took place in December while the main peak occurred during the New Year’s Eve (Figure 5f).

We apply our climate-dependent multiple-regression model to the forecast period (November 19, 2020, to February 2, 2021), in order to predict the infection index for each health area (*I_c,pred_*) during the pandemic’s third outbreak (Figure 5). Despite differences in amplitude, most BHAs illustrate three peaks in *I_c_* during mid- and end-December 2020 and in January 2021 (red lines, Figure 5). Outstandingly, the predictions reproduce the basic features in the observed index (*I_c_*) for most BHAs (light orange lines, Figure 5). In particular, the model reproduces the increase of *I_c_* during mid-December and January in LLEI-2 (*r* = 0.33), SJD (*r* = 0.42), GVA-2 (*r* = 0.40) and BCN-10A (*r* = 0.33) (Figures 5a–c,g). Notice that for GVA-2 and BCN-10A the climate-based model simulates the enhancement of the infection rate 5–7 days earlier than in the observations (Figures 5a,b). However, the model generally fails to reproduce the *I_c_* peak at the end of December. This suggests that this peak may have been largely driven by the relaxation of social restrictions during the Christmas festivities. For the remaining BHAs (TRG-2, TRS-E and RUB-3), the correlation remains significant (0.40 to 0.47) when only the second half of the period (December 25 to January 20) is considered (Figures 5e,f,h). However, the model fails completely to forecast *I_c_* in SVH-2 for the entire period, showing even an out-of-phase behavior between the observed and predicted values (Figure 5d). The same behavior is also recognizable when the observations in SVH-2 are compared to the observed values for the other areas (e.g., BCN-10A, GVA-2 or SJD).

The relative success of the climate model is remarkable if we consider that the forecast included periods of limited mobility and social interactions, with temporary relaxation during the holiday seasons. Finally, it is important to note that when the normalized number of cases is low then the observed and predicted infection indexes show no significant correlation (*p* ≥ 0.01), as it also happens in several BHAs between November 19 and the end of the year (Supplementary Figure S6).

## 4 Discussion

We have explored the spread of SARS-CoV-2 infection in Catalonia (northwestern Mediterranean) for periods when there were limited mobility and social restrictions and there was not yet a vaccine available. In this way we have minimized the effects associated with decreased SARS-CoV-2 transmissivity related to acquired immunization and lockdown-mobility restrictions in areas with high-density population (38). Our results show significant correlations of both surface pressure (*P*) and relative humidity (*RH*) with the daily infection index (*I_c_*), at ɑ = 1% and ɑ = 5%, respectively, in all basic health areas (BHAs); solar radiation (*Rad*) and daily thermal amplitude (*DTA*) are also significantly correlated with *I_c_* (*p* < 0.05) but only in some BHAs while consistent correlation does not exist between the other meteorological variables evaluated and *I_c_*. This allows building a simple multiple linear regression model for the infection index with only two predictors, *P* and *RH*; in this model, the regression coefficients for *P* and *RH* are negative for all BHAs studied, indicating that a decrease of *P* and *RH* causes an increase of the *I_c_* index after several days. The regression model has predictive capacity with a significance level below 5% for the setup period. Remarkably, the cross-correlation of *I_c_* and either *P* or *RH* provides consistent and significant results, strongly suggesting that *P* and *RH* lead the COVID-19 outbreaks by 7 days and 3 days lag, respectively.

Outstandingly, our climate-dependent model shows a moderate skill to forecast the SARS-CoV-2 transmission during a later period despite real limitations, such as the accuracy of the daily counts of COVID-19, and potential restraints, such as the interrelation between weather variables. The predictive skill of our model is significant at a 90% level in four out of the eight BHAs (BCN-10A, GVA-2, SJD and LLEI-2), and remains significant during shorter periods for three of the other areas (RUB-3, TRS-E and TRG-2; correlation scores between 0.33 and 0.47). This means that weather conditions are able to explain between 14 and 45% of the variability of the infection index *I_c_* in the selected eight BHAs of Catalonia during the setup period (September 1 to November 15), and between 11 and 22% during the predictive period. The reduction in this latter period is likely related to the changing social interaction and mobility measures, much greater than during the setup period.

Our findings are also consistent with the results of other studies, in particular those conducted in the Iberian Peninsula. Fernández-Ahúja and Martínez (39) found that surface pressure and infection cases are negatively correlated at a regional scale. Sanchez-Lorenzo et al. (40) suggested that dry conditions favor the spread of the virus during the initial stage of the pandemic. No association between infection cases and mean daily temperature was found in another study in Catalonia (90). Our results corroborate these findings on a smaller spatial scale during other pandemic waves and using a different approach, i.e., a MLR analysis with time lags.

The mechanisms for weather-mediated changes in respiratory disease include the effects of weather on virus survival in the air and surfaces of outdoors/indoors spaces, changes at the individual-level susceptibility toward the disease, and also variations in social human behavior. The 7-days lag between a decrease in surface pressure and the onset of a peak of infection agrees with the incubation period of the SARS-CoV-2 variants circulating during the study period [mean of 5.7 days and a range between 2 and 14 days; (41)]. The predictor capacity of surface level pressure on the virus expansion may possibly arise from both direct and indirect causes. A pressure change is the main indicator for the passage of low- and high-pressure frontal systems that bring substantial changes in weather, such as temperature, precipitation or wind velocity. Further, rapid changes in weather conditions may affect the susceptibility to airborne virus infection with disruption of local mucosal immunity. An indirect effect may be the weather-related changes in human behavior, with the most evident response under bad weather conditions being to remain indoors, where the virus can persist longer. In enclosed spaces with inadequate ventilation, small infected droplets and particles can remain suspended from minutes to hours (42, 43). Furthermore, the chances of close contacts increase hence leading to an enhanced virus spreading capacity (44–46). Indeed, it is widely accepted that most of the infections occur in indoor spaces, frequently as super-spreading events (47–49). A scientific report led by the WHO-China commission concluded that 78–85% of transmissions occurred within household settings during the first wave, indicating that transmission is likely to occur during close and prolonged contact scenarios (92). Further, the outdoor chains of transmission, which are much more influenced by direct weather conditions, also define the chances that infected individuals go indoors and provoke super-spreading events (50).

The 3-days lag for relative humidity is more difficult to justify, even if it still lies between the estimated incubation bounds. The linear model predicts that dry conditions will favor the propagation of the virus (an increase in *I_c_*) about 3 days later and wet conditions will tend to inhibit it. Low humidity, indicative of dry weather, has been identified as a key factor associated with the transmission and stability of respiratory viruses such as influenza (51). Dry weather conditions enhance the susceptibility and severity of influenza infection through disruption of local mucosal immunity of the respiratory tract (35, 51–53); additionally, they increase the stability of the SARS-CoV-2 in the environment (54, 55). Accordingly, some studies support an inverse relationship between humidity and the spreading of SARS-CoV-2, consistent with our findings (56, 57). However, a positive effect of relative humidity toward SARS-CoV-2 infectiousness has also been found in other studies (58–63); in particular, a recent study in England and Wales has found that coronavirus have a different pattern of weather susceptibility as compared with the influenza virus, with an increase of transmission (above 80%) during periods of high relative humidity, which behaves as a better predictor than specific or absolute humidity (64).

The disparity of findings related to humidity could be explained by the convex relationship between virus stability and humidity, which is highest under both high and low *RH* (54). It also suggests that there may be geographically-dependent factors that modulate local humidity conditions and the virus response (51). Several studies suggest that dry conditions may have favored the fast spread of the virus in Spain at the onset of the pandemic (40, 65), although there is no full consensus (66). A recent review underlines the significant association between geographic factors (e.g., location, demography) and SARS-CoV-2 spreading at regional or local levels, with the potential interplay of meteorological factors (67). Future studies should consider the succession of weather episodes that can foster or inhibit virus expansion and their interaction between outdoors and indoors conditions (51), e.g., episodes of dry weather that favor environmental outdoors stability of the virus followed by rainy episodes that increase the chances of indoors transmission.

Our results show that surface pressure is the major weather driver of the SARS-CoV-2 spread, and indeed this is probably the main atmospheric indicator for the arrival of frontal systems. Depending on latitude and location – e.g., west versus east coasts of continents – these systems will typically arrive from different directions and cross either land (dry) or sea (wet) regions, hence driving a decrease or an increase in humidity. This idea fits with our finding that surface pressure is the main weather parameter influencing the spreading of the virus while relative humidity plays a more secondary role. Something similar could happen with other possibly secondary variables such as sunlight radiation, which we observe to have significant correlations with the infection index in some BHAs and has been related to SARS-CoV-2 and other airborne viruses (68).

According to a recent comprehensive review (69), temperature is the weather variable that bears major relation with the different infection variables indicative of SARS-CoV-2 spread (number of confirmed COVID-19 cases, number of COVID-19 death cases, cumulative incidence, clinically compatible or PCR based diagnostic cases, among others), with warm and wet climates generally (but not always) decreasing COVID-19 incidence. This same review also shows that high humidity is generally associated with higher incidence/prevalence of the infection but with substantial variability. This review also identifies that surface pressure in some cases is correlated to the occurrence of COVID-19, but this correlation appears much less frequent than temperature or humidity (69).

Models based solely on atmospheric variables have failed to predict the incidence of the disease probably due to the presence of other factors beyond the interrelations between different weather variables. These include: an incomplete consideration of relevant social variables, such as the mobility and distancing restrictions imposed during the succeeding SARS-CoV-2 waves; the diverse infection outputs and inconsistencies in the counting system of infected population (70); and the broad range of indexes used to measure the epidemic activity. In our case, the absence of a stagewise analysis could be considered a limitation (71), as different events, such as the start of the school year in mid-September and the Christmas holidays, might have influenced infection dynamics. However, this limitation is only relevant during the Christmas period due to the substantial increase in social interactions. Schools, on the other hand, did not significantly contribute to transmission among children, as the circulating strains at the time had minimal impact on this group (98–100). Moreover, the infection index *I_c_* used in our study provides a robust estimate of SARS-CoV-2 activity, as it is based on direct, systematic laboratory-based screening at the community level.

It is important to emphasize that the primary aim of our study has been to show that weather conditions, as reflected by a selected number of variables, do have an effect on the spread of COVID-19. The objective was not to obtain good fits but rather to identify atmospheric factors that do affect COVID-19 infections. From this perspective, our results show that surface pressure and relative humidity do have a significant influence on epidemic outbreaks. Nevertheless, the relatively low correlation between these weather variables and the infection index confirms that the lack of population immunity remains as the primary driver, with high susceptibility associated with the absence of naturally acquired immunity, the low effectivity of vaccination against infection, and the limited vaccine coverage (72). The inability of current vaccines and past infections to produce a long-standing immunity, and the definitive leverage of most of the social restrictions by the end of 2022, underscore the importance of understanding the interplay between weather conditions and the epidemiological dynamics of COVID-19. In this post-pandemic induction phase, weather conditions may substantially affect the onset and extent of new waves and outbreaks as observed in other respiratory viruses. This study illustrates the potential of linear multiple-regression models as a useful tool to identify the right combination of weather variables that preconditions the development of respiratory pathogens and pandemics.

## Data Availability

The datasets analyzed for this study can be found in the Catalan Transparency Portal databases for each data set: automatic weather stations (XEMA) from the Meteorological Service of Catalonia, https://analisi.transparenciacatalunya.cat/Medi-Ambient/Dades-meteorol-giques-de-la-XEMA/nzvn-apee, and open database of COVID19 record from the Health Department of Catalonia, https://analisi.transparenciacatalunya.cat/Salut/Registre-de-casos-de-COVID-19-a-Catalunya-per-rea-/xuwf-dxjd. The datasets are also available from the corresponding authors on reasonable request.
