# Supplementary material for: Environmental predictors of SARS-CoV-2 infection incidence in Catalonia (northwestern Mediterranean)
Source: Front Public Health. 2024 Dec 5;12:1430902. doi: 10.3389/fpubh.2024.1430902 (PMC11656081; doi:10.3389/fpubh.2024.1430902)
Supplement: Supplementary file 1 [file Data_Sheet_1.pdf]

Supplementary information for

Environmental predictors of SARS-CoV-2 infection incidence in Catalonia (northwestern Mediterranean)

Jesús Planella-Morató[1,2,3], Josep L. Pelegrí[1], Marta Martín-Rey[4], Anna Olivé-Abelló[1], Xavier Vallès [5,6,7], Josep Roca[8], Carlos Rodrigo[9,10], Oriol Estrada[11], Ignasi Vallès-Casanova[1,12,13]

1. Departament d'Oceanografia Física i Tecnològica, Institut de Ciències del Mar, CSIC, Barcelona, Spain
2. Departament de Físicca, Universitat de Girona, Girona, Spain
3. University School of Health and Sport (EUSES), University of Girona, Girona, Spain
4. Departamento de Física de la Tierra y Astrofísica, Universidad Complutense de Madrid, Madrid, Spain
5. Fundació Lluita contra les Infeccions, Badalona, Spain
6. Fundació Institut per la Recerca Germans Trias i Pujol, Badalona, Spain
7. Programa de Salut Internacional Institut Català de la Salut (PROSICS), Badalona, Spain
8. Epidemiology Unit, Hospital Universitari Germans Trias i Pujol, Institut Català de Salut, Badalona, Spain
9. Department of Pediatrics, Institut de Recerca Germans Trias i Pujol, Badalona, Spain
10. Universitat Autònoma de Barcelona (UAB), Barcelona, Spain.
11. Directorate for Innovation and Interdisciplinary Cooperation, Northern Metropolitan Region from Barcelona, Institut Català de la Salut, Barcelona, Spain
12. Hebrew University of Jerusalem, Jerusalem, Israel
13. Centro Oceanográfico de Santander, Instituto Español de Oceanografía, Santander, Spain

Weather characterization and main events during the COVID-19 second wave

In the first half of September, 2020, the highest precipitation event for the period that includes the second wave (September 1 to November 15, 2020) was recorded in most of the basic health areas (BHAs) considered. On September 6, a humid and cold front entered Catalonia from the northwest, following east with 100% relative humidity, low temperatures and leaving high rainfall in the province of Girona and in the coast and pre-coastal of Barcelona province. On September 7, the front crossed south the region, causing heavy precipitation in all BHAs high relative humidity (65 – 80%), low solar radiation (35 - 60 MJ m^{-2}) and a pressure around 1019 hPa. On September 8, the front moved eastwards into the Mediterranean leaving no precipitation, a drop in relative humidity to less than 60%, an increase in surface atmospheric pressure, and a daily thermal amplitude of about 10 ºC in all coastal BHAs. Finally, on September 9, the front moved along the Catalan coast with very high relative humidity (75 - 85%), lowering the daily thermal amplitude and the solar radiation to 3 ºC and 18 MJ m^{-2}, respectively, in those BHAs near Barcelona (Figures S1 and S2).

On September 18 and 19, 2020, another front entered from the east, leaving notable precipitation in all BHAs up to 20 mm, mostly those in the interior areas. The relative humidity was 80% close to Barcelona, daily thermal amplitudes of 4 ºC on the coast and around 9.7 ºC in Lleida, and fairly homogeneous surface pressure near 1014 hPa. On October 2 and 10, precipitation appeared again. On October 2, minimum radiation, daily temperature variation, surface pressure and relative humidity recorded maximum values in most BHAs. Surface pressure and relative humidity gradually increased in the successive days until reaching a maximum on October 10, with the heaviest precipitation and highest relative humidity for the entire month. On October 14, there was again an event of low temperature, significant precipitation, low solar irradiation, high humidity (≥ 60%) and daily thermal amplitudes of 7ºC near Barcelona. Finally, by the end of the second wave, from November 3 to 7, a precipitation front entered from the north, generating widespread intermittent precipitation throughout Catalonia, coinciding with a remarkable minimum of solar radiation down to 100 MJ m^{-2} and surface pressure around 1024, thermal amplitude about 2 ºC and relative humidity between 70 and 96%.

Figure S2 shows, for each BHA, the time series of all eight climatic variables considered in our study along with the normalized number of positive cases of COVID-19, $\widehat{N}_{PCR,+}$, and the infection index I_c. The details of the evolution of I_c and $\widehat{N}_{PCR,+}$ during the second wave have been included in the main text for all BHAs. For BHA BCN-10A, this information has been complemented with a brief weather description according to the information provided in this section.

Time lags between infection index and weather variables

Figure S3 shows the dependence of the cross-correlation coefficients between the infection index and the weather variables as a function of time lag, in days, for all eight BHAs (BCN-10A, GVA-2, SJD, SVH-2, RUB-3, TRS-E, LLEI-2 and TRG-2).

Model parameters and statistics

The analysis of the cross-correlated coefficients (*CCF*) indicates that only the surface pressure *P* and the relative humidity *RH* have *CCF* below a threshold of α = 5% at all the BHAs (Figure S3). Therefore, only these two weather variables have been considered as potential predictors to build the model (see Methods). After this first selection of candidate variables, the propagation of the virus is modelled using a multiple linear regression (MLR) on those predictors, as described in Eq. (3) (see Methods). The collinearity between the two predictors has been inspected and the results of the corresponding tests are shown in Table S1. The variables have weak positive correlations ($r_{P,RH}$), with values that range between 0.2 and 0.4. However, in all BHAs, this correlation is statistically significant ($p_{TS} \leq 0.1$), indicating that the collinearity effects may not be disregarded. Hence, the relationship between *P* and *RH* has been also analyzed through the variance inflation index (*VIF*) of the two predictors used in the model. It is found that the values of *VIF* are less than 2.5 for all areas, which is a conservative threshold suggested in literature. We conclude that, although collinearity between the surface pressure and the relative humidity could exist, the effects on the model parameters are not severe and they can be considered as independent variables.

After the inspection of the collinearity between candidate predictors, the hypothesis tests have been applied to the coefficients of the model, in a joint and separate way. If we test the model as a whole, the joint test inspects whether the MLR provides a better fit to the observations than a model that contains no predictors. The results of the joint *F*-test are shown in Table S2. Based on these results, we can conclude that there is strong evidence that at least one of the two climatic variables is linearly related to the observations in all BHAs ($p < 0.01$). However, this does not mean that the two predictors must be included in the model to explain this relationship.

In addition, the intercept and the regression coefficients for the model at each BHA and their corresponding statistical parameters are listed in Tables S3, S4 and S5. Table S3 indicates that the regression coefficient for the surface pressure is not significant ($p > 0.1$) in the BHA of TRG-2. On the other hand, the relative humidity is not significantly correlated with the infection index in the BHAs of GVA-2, SVH-2 and TRS-E ($p > 0.1$). The results indicate that the model used in four BHAs (BCN 10-A, SJD, RUB-3 and LLEI-2) includes two predictors (surface pressure and relative humidity) but only one predictor in the remaining BHAs (surface pressure in GVA-2, SVH-2 and TRS-E and relative humidity in TRG-2).

To corroborate the previous results, we have also tested the addition of predictors using the stepwise estimation method applied forwards. The results for each BHA are shown in Table S6. In all stations except TRG-2, the process starts with the surface pressure, which is the predictor that holds the highest correlation coefficient with the infection index. The critical value of the partial *F*-statistic, F^{*}_{par} at $\alpha = 10\%$ is 2.78. The F_{par} values reveal that the addition of the relative humidity is not significant in GVA-2, SVH-2 and TRS-E, so it is removed from the final model. On the other hand, in TRG-2, the model does not improve when the surface pressure is added and it is hence removed from the final model. These results are in agreement with the results obtained from the analysis of the statistical significance of the regression coefficients. To summarize, we conclude that the final model includes two predictors that have significant coefficients ($p < 0.1$) in four out of the eight health areas. In the other four areas, only one predictor would have a significant contribution to the final model (three areas for the surface pressure and one for the relative humidity).

The infection index has been used to fit the data to the model and obtain the estimates of the parameters $\forall c_i$, $i \in [0,2]$ of Eq. (3) (see Methods). Following the results of the partial *F*-test, some of the regression coefficients, either c_1 or c_2, are set to 0 in several health areas. Table S7 summarizes the regression coefficients in the final model and several goodness-of-fit parameters used to describe how well the linear regression fits the corresponding model in each BHA, such as the standard R-squared (r^2) and its adjusted coefficient (r^2_{adj}). The analysis of the coefficients listed in this table shows that the values of the coefficient c_1 are statistically significant ($p < 0.01$); the values of c_1 vary within $[-10, -3]\cdot10^{-3}$ hPa^{-1} (mean(c_1) = $-5.90\cdot10^{-3}$ hPa^{-1}; std.(c_1) = $1.90\cdot10^{-3}$ hPa^{-1}). The values of c_2 are also significant ($p < 0.05$) and range between $-4\cdot10^{-3}$ and -1×10^{-3} (mean(c_2) =$-1.91\cdot10^{-3}$; std.(c_2) =$1.12\cdot10^{-3}$). The intercept c_0 is found to be significant ($p < 0.05$) in all BHAs except TRG-2 ($c_0 \in (0.4, 10)$; mean(c_0) = 6.15; std.(c_0) = 1.82).

As the forward stepwise method used in our model has some bias, we have built another model for each BHA using the unbiased two-step approach (Hosmer and Lemeshow, 2013). The effect of the bias in our model has been assessed by comparison between the two models. The new model is built shifting each variable according to its characteristic time lag (cross-correlation analysis) and testing with a univariable analysis. In order to select the candidate predictors, a *p* value of 0.25 has been chosen as an adequate cut-off for statistical significance (Hosmer and Lemeshow, 2013; Zhang, 2016; Kebalepile and Chakane, 2023). Those variables that turn

statistically significant ($p < 0.25$) are included in the model. A second step consists of fitting the MLR model, which includes all the variables with regression coefficients identified as statistically significant, to the data. Finally, the forward stepwise model is compared to the two-step approach by calculating the percent change Δc_i in the regression coefficients for surface pressure and relative humidity. If the coefficients change less than 20%, the deleted variables do not have a relevant impact on the regression coefficients of the remaining variables, which are hence removed from the model.

The results of the univariable analysis for the eight BHAs are shown in tables S8 to S15. The files corresponding to weather variables with non-significant regression coefficients ($p < 0.25$) are highlighted in grey. The specific analysis performed at each BHA shows that surface pressure and relative humidity are significant in all health areas and their regression coefficients show, in general, the smallest *p* values. In contrast, the *p* values of the coefficients of the other weather variables are often below the threshold for statistical significance, in many health areas. For example, the *p* values for the regression coefficients of solar radiation and precipitation are lower than the cut-off value in seven out of the eight BHAs, but for the daily temperature average (*DTA*) they are only statistically significant in three BHAs.

The regression coefficients of the surface pressure and the relative humidity obtained from the two-step model are compared with the coefficients obtained from the forward stepwise approach (Table S16). The results indicate that the percent variation is less than 20% for all the coefficients, varying between a maximum difference of 19.6% for surface pressure at GVA-2 and a minimum difference of 1.8% for relative humidity at LLEI-2. According to the coefficient variation threshold ($\Delta c_i < 20\%$), we conclude that the weather variables excluded in our model do not cause a substantial change in the regression coefficients of both surface pressure and relative humidity. Then, the bias introduced by the method in the model can be neglected and a model based only on surface pressure and relative humidity fits reasonably well the data.

The residuals of the regression adjustment have also been analyzed to investigate other relevant assumptions in the linear models. The assumption of normality has been inspected from the probability plots of the standardized residuals (Figure S4). In some BHAs, the plots reveal the presence of long upper tails in the empirical distributions, which differ substantially from the expected normal distributions. These tails are easily identified in the probability plots for the residuals obtained in SJD, SVH-2 or TRG-2. The Kolmogorov-Smirnov (K-S) test has been

performed to investigate whether the deviations from normality observed in the probability plots are significant or not. The results of the K-S test (Table S17) show that the normality of the residuals is satisfied at α = 1% in all BHAs except in TRG-2. On the other hand, the White test for heteroscedasticity indicates that the variance of the errors is constant (null hypothesis is not rejected) in all health areas. The deviations observed in the tails and the use of low significance levels in the heteroscedasticity tests suggest some potential problems in the collection of samples in some BHAs; indeed, it is known that data reporting was a relevant issue during the early waves of the pandemic.

Internal validation of the model setup during the pandemics second wave

The predictions for the infection index in each BHA have been estimated using the MLR model described in Eq. (8) (see Methods), with the coefficients c_0, c_1 and c_2 in this equation replaced with the corresponding values listed in Table S7. The results obtained in each BHA (n = 72) are shown in Figure S5, where the grey circles indicate the observed values I_c represented against the predictions $I_{c,pred}$. The dashed line corresponds to the regression line between the two variables, I_c and $I_{c,pred}$. As expected from the model, the slope and the y-intercept of the linear regressions are equal to 1 and 0 respectively, and both are statistically significant (the t-statistic for these parameters is close to 0).

Figure S5 also includes data of the validation performed internally using the leave-one-out cross validation (LOOCV) method. In most of the BHAs, the results of the LOOCV method show that the cross-validated $I_{c,[i]}$ values fall close to the predictions $I_{c,pred}$ obtained from the model. Concretely, these values are very similar for small and moderate values of the infection index ($I_c \in [0.05, 0.15]$). However, if very small and large values of I_c are considered, the cross-validated $I_{c,[i]}$ indexes deviate substantially from their corresponding predictions $I_{c,pred}$. In general, if the value of I_c is large, the model underestimates the real value and the cross-validated value is lower than prediction. On the other hand, the model has the opposite behaviour for very small values of I_c, indicating possible model overestimation. This agrees with the deviations from normality observed in the upper tails of the probability distribution plots of the residuals, especially in SJD, GVA-2 and TRG-2 (Figure S4). For SVH-2, the differences between $I_{c,pred}$ and $I_{c,[i]}$ are in the range of observations. This also agrees with the probability plot of the residuals obtained in this area. Thus, Figure S5 suggests that the model has some limitations to predict adequately the highest and lowest values of the infection index. This could be related to the absence of relevant variables in the model, such as mobility factors,

which can impact on the predictions in situations when these factors play an important role. Additionally, the particular situations in each BHA could also affect the timely report of positive cases. Finally, the linear model can experience some difficulties to properly capture sudden changes in the observations.

The differences between the observed and predicted values at each BHA have been evaluated via the absolute percentage error APE_i of each measurement. These errors have been grouped into 10% intervals in the range from 0 to 100%, grouped into a single interval for APE_i > 100%. To validate the model, the same procedure has been applied to the cross-validation errors ($APE_{[i]}$). The results obtained for each BHAs are displayed in the multiple charts insets of Figure S5, showing the prediction errors using both the model and the cross-validation.

The relationship between the observed and predicted values has been assessed via the regression analyses. Table S18 includes the estimates of several statistical parameters for the model and the cross-validation, including R-squared, *MAPE* and *RMSE*. The two values are compared in order to test the overall performance of the model. These results are complemented with the analysis of the y-intercept (β_0^{CV}) and the slope (β_1^{CV}) of the linear regression between the observed and cross-validated values (Table S19) in order to check whether they differ significantly from the expected values ($\beta_0 = 0$ for the y-intercept and $\beta_1 = 1$ for the slope). The error sources can be associated with the mean differences between observed and predicted values (bias), the deviations from the 1:1 relation (slope) and the unexplained variance. The significance tests for $\beta_0^{CV} = 0$ (bias) and $\beta_1^{CV} = 1$ (dispersion around the 1:1 relation) allows assessing the predictive skill of the model based on the results of the cross-validation, that is, if the model is consistent (unbiased and with small differences between observations and predictions).

The model has been validated for all BHAs through the relevant statistical parameters in Table S19. For example, the R-squared q^2_{CV} of the cross-validation is obtained by linear fitting the observed I_c and the validated predictions $I_{c,[i]}$. The q^2_{CV} values are statistically significant (p < 0.01) for all BHAs, ranging between about 0.10 and 0.40. In general, the values of q^2_{CV} are similar to those R-squared (r^2) values obtained by fitting the observations to the model predictions, with differences of about 10-15% (see Table S18). These results support that a weak but significant linear relationship exists between the observations and the weather variables. Table S19 also includes the values of the y-intercept β_0^{CV} and the slope β_1^{CV} of the

cross-validations for each BHA, and the p values of their corresponding significance tests, for $\beta_0^{CV} = 0$ and $\beta_1^{CV} = 1$, respectively. For all BHAs, the slopes are slightly below the 1:1 line with values oscillating between 0.84 and 1. However, the significance of the tests for β_1^{CV} indicates that the slope is not significantly different from 1 ($p > 0.01$, i.e. the null hypothesis H_0: $\beta_1 = 1$ cannot be rejected at α = 1%). On the other hand, the intercept for all BHAs is found to be positive and near zero, with values between about 0.01 and 0.04 for all the areas except SVH-2, where the value is 0. Also, the p values associated with the t-statistic of the significance tests for β_0^{CV} show that the y-intercept in the linear regressions (of all BHAs) does not differ significantly from 0 ($p > 0.01$, then H_0: $\beta_0 = 0$ is not rejected). The results of the cross-validation also suggest that most of the errors in the model predictions are related to the unexplained variance, hence not caused by any bias trend or misleading slope. We conclude that the model is consistent and unbiased but not accurate because the unexplained variance can cause substantial errors in the predictions.

The errors in the predictions have been investigated in more detail based on other statistical parameters, such as the mean absolute percentage error (*MAPE*) and the root-mean-square (*RMSE*), and have been validated via the LOOCV method (Table S18). The *MAPE* and the *RSME* model prediction errors agree with those obtained using cross-validation ($MAPE_{CV}$ and $RMSE_{CV}$). The *MAPE* values exceed 50% in five of the eight BHAs, reaching over 100% for TRG-2; this is also observed from the cross-validated results (Table S19). However, for all BHAs the mean prediction errors ($MAPE_{CV}$) of the cross-validation do not deviate more than 5% with respect to the corresponding model values (*MAPE*). These results are consistent with the *RMSE* estimates in all BHAs, also validated using the LOOCV method (differences between *RMSE* and $RMSE_{CV}$ are also ≤ 5%). The BHA with the highest *RMSE* value is TRG-2, which also exhibits the highest *MAPE*. This trend is observed in most of the BHAs analysed in this study and is visually clear in Figure S5. For example, the TRG-2 data have the largest dispersion around the best-fit line, supporting that both the *MAPE* and *RMSE* values are highest for this health area.

For each BHA, the variability observed in the predictions with respect to the observations is discussed in more detail based on the percentages of the absolute prediction errors (*APE*) and assessed via cross-validation. This procedure also allows investigating the possible error sources in the model predictions. In all BHAs – except for SJD, TRG-2 and SVH-2 – an important fraction of the data (≥ 45% of the data) have relatively small *APE*s in the predictions

(APE < 30 %) (Figure S5). The highest percentage (about 60% of the data) is found in BCN-10A and TRS-E. Note that BCN-10A, LLEI-2, RUB-3 and TRS-E exhibit relevant percentages of data (about 35%) with APE < 20%. On the other hand, SJD, SVH-2 and TRG-2 have smaller amount of data (≤ 40% of the data) with small errors (APE < 30%). The lowest percentage of data (about 25% of the data) is found in TRG-2. Focusing on the large percentage errors, these three last BHAs show values of APE > 60% in over 30% of data and, once again, the worst percentage is obtained for TRG-2, where about 35% of the data concentrate the largest errors (APE > 100%). For the other five BHAs, the amount of data with large errors (APE > 60%) are smaller (< 30%), decreasing further for GVA-2 and LLEI-2 (about 20%). The minimum percentage of data with large errors is reached in BCN-10A, with about only 12%. The results are validated computing the absolute percentage errors (APE_{CV}) of the cross-validation (Figure S5). It is found that the APE_{CV} values have distributions similar to those obtained from the model, and the differences between the relative frequencies of APE and APE_{CV} is less than 5%. The slope and y-intercept of the fittings explain the $MAPE$ and $RMSE$ differences in the model and cross-validated values for all BHAs (Table S18).

External validation of the model forecast during the pandemics third wave

The meteorological and health data for the period from November 19, 2020, to February 28, 2021, have been inspected to validate the model externally. Figure S6 shows the time evolution of the normalized cases $\widehat{N}_{PCR,+}$ in this time period for all BHAs.

The pandemics third wave is clearly visible in most of the BHAs (Figure S6). In general, the plots reflect a rapid growth in COVID-19 cases during December, reaching their highest peak in January, followed by a substantial reduction of infections to low levels at the beginning of February. Note that SVH-2 has a more irregular behaviour, with two narrow peaks, one located around mid-December and another at the end of January. Also, LLEI-2 displays a different pattern, with no clear wide peaks. Although the time period until late February 2021 could appear as appropriate to externally validate the model, some of the BHAs show normalized number of cases ($\widehat{N}_{PCR,+}$) close to 0, which may lead to spurious predictions of the daily infectious index. Therefore, for some BHAs, such as TRG-2, TRS-E or RUB-3, the model has been validated using subsets of data that include representative $\widehat{N}_{PCR,+}$ values; in TRS-E and TRG-2 this period of time is relatively short (Table S20). Finally, we have computed the correlation between the predicted and observed values of the infection index, and the external

validation of the model has been performed for the data subsets that exhibit the highest correlation.

The external validation of the model follows the same method implemented for the internal validation and described previously (see Methods). For each BHA, the infection index I_c was computed based on the normalized number of positive cases, $\widehat{N}_{PCR,+}$, and Eq. (8) (see Methods) was applied in order to obtain the predicted values for the new observations I_c. Finally, the predicted values ($I_{c,pred}$) were used to validate the model externally during the pandemics third wave.

Figure S7 shows a scatter plot of observations against the corresponding predictions. The inset bar charts represent the relative frequency of the absolute prediction errors (*APE*'), which are expressed in percentage and grouped in 10% intervals. The plots exhibit a positive correlation between the observed I_c and predicted $I_{c,pred}$ values in all the BHAs except SVH-2. In this latter area, the data points appear to be randomly scattered, indicating no correlation between I_c. and $I_{c,pred}$. This is corroborated by the values of the predicted R-squared (r^2_{ext}) obtained from the regression fits, which are represented as solid lines and displayed along with their corresponding linear equation in each panel of Figure S7 (see also Table S12). Although the data are uncorrelated ($r^2_{ext} \cong 0$) for SVH-2, a weak positive relationship between observations and predictions is found in all other BHAs, with r^2_{ext} between 0.1 and 0.2. The F-test of the linear-prediction overall significance has been conducted to confirm the strength of this relationship.

Table S20 evidences a linear relationship between the observations and the model predictions ($p < 0.01$) in four of the BHAs (BCN-10A, GVA-2, SJD and LLEI-2), which are those with the largest amount of data. If the size of the sample decreases, the evidence weakens but it is still moderately significant ($p < 0.05$), suggesting that the results obtained in small size datasets should be considered with caution. On the other hand, the amount of data for SVH-2 is large but the result of the test indicates that there is no significant linear relationship between observations and predictions ($p > 0.1$). In this case, $\widehat{N}_{PCR,+}$ behaves more irregularly as compared with other BHAs (Figure S7). This may explain the lack of skill of the model to capture the main features of the virus propagation in this area, suggesting that the variability of the observations is largely attributed to factors other than the weather conditions.

For the BHAs with a significant linear relationship – all but SVH-2 – the parameters of the linear equations are analyzed in more detail (equations displayed in Figure S7). The linear regressions lie below the 1:1 line and the y-intercept is a positive value close to zero. There appears to be a tendency of the model to overestimate the predictions of the infection index, in particular for GVA-2, RUB-3 and TRS-E. In these cases, the errors in the predictions associated with bias or lack of model consistency could be significant. In order to evaluate this possibility, the values of the slope (β_1^*) and the y-intercept (β_0^*) of the linear fits have been checked by performing their corresponding tests of significance. The results of the tests (Table S20) show that, in all BHAs except SJD, the values of both parameters do not differ significantly from their expected values, $\beta_1^*= 1$ and $\beta_0^*= 0$ respectively ($p > 0.01$, i.e. the null hypotheses H_0 of each test is not rejected at $\alpha = 1$ %). As a result, we can conclude that, for six out of the seven health areas analysed, there is not enough statistical evidence to infer that the model is biased ($\beta_0^* \neq 0$) or inconsistent ($\beta_1^* \neq 1$). This is in agreement with the results obtained from the internal validation and suggests that the unexplained variance may be caused by factors other than weather. Only for SJD the results of the significance tests show that the values of the slope and the y-intercept are significantly different from the expected values, suggesting that the model tends to overestimate the predictions in this health area. The weaker consistency of the model predictions in SJD, and the deviation from normality in the upper 20% of the residuals probability plots (Figure S4), suggest that there may have been some infection reporting issues in this area.

Finally, the prediction error statistics have also been computed for each BHA. It includes the $MAPE$ and $RMSE$ values for external validation along with the predicted R-squared (r_{ext}^2) of the regression and its adjusted version ($r_{ext,adj}^2$) (Table S21). The $MAPE$ value is above 50% in six out of the seven analysed BHAs and exceeds the 100% error in LLEI-2 and TRG-2. In general, the values found for the external validation are about 40% to 60% higher than the corresponding values used to build the model (Table S18). This can be explained by the presence of a higher percentage of data with large errors in the predictions when the model is validated externally. The bar charts inserted in the Figure S7 show that, in most of the areas, about 30 to 40% of the data have percentage absolute errors (APE') higher than 60% with the largest errors (APE' > 100%) observed in 20 to 30% of the dataset. The worst results are found in LLEI-2 and TRG-2, where about 60% of the data have APE' > 60%. In general, the data percentages differ by 10 to 15% from those obtained when the model was built (see Table S18). An exception is found in TRS-E, where the large-error percentages remain almost the same in

both situations. The analysis for the small errors reports similar results. If the external validation is applied, the percentage of data with *APE*' < 30% decreases down to around 30% in most of the BHAs except for BCN-10A, GVA-2 and TRS-E, where the data percentage remains at about 45%. In these areas, about one third of the data still contains the lowest errors (*APE*' < 20%). Although the percentages of data with small errors are relevant, they are 10 to 15% lower than those obtained in the model setup period (Table S18). The changes in the distribution of the large and small errors in the predictions explain the differences in the absolute-prediction-error averages between the setup and forecast periods. Notice that the health areas with the lowest averaged prediction errors are the same, independently of the time period analysed (see BCN-10A or TRS-E, for example). This is also observed for the health area with the highest averaged prediction error (TRG-2), although the error substantially increases for LLEI-2 when the external validation is applied.

The *RMSE* from the model is also calculated for each health area and the forecast periods. In general, the *RMSE* differences between the forecast and setup periods are considerably lower than those obtained with the *MAPE* analysis. In four out of the six BHAs, the values obtained from the external validation increase by 5 to 10% with respect to those values obtained when the model was built. This percentage is larger in BCN-10A (about 35%) and reaches its maximum value for LLEI-2 (80%). In TRG-2, the *RMSE* value is the highest but decreases as compared to the model setup period (Table S18). For the external validation, the r_{ext}^2 coefficients vary between 0.1 and 0.2 and decrease further when the modified coefficient $r_{ext,adj}^2$ is considered. These values are generally lower than those obtained from the dataset used to build the model (Table S18). Although the data are significantly correlated in both the setup and forecast scenarios, the linear relationship weakens when the external dataset is used. However, a percentage of the infection-index variance during the pandemics third wave can be still explained by the predictors used in the model (pressure and/or relative humidity).

References

Hosmer, D. W., Lemeshow, S., and Sturdivant, R. X. (2013). Applied logistic regression. Hoboken, New Jersey: John Wiley and Sons.

Kebalepile, M. M., and Chakane, P.M. (2023). Regression analysis basics: making the right choice of type of regression analysis to model clinical data. South. Afr. J. Anaesth. Analg. 29(01), 136-142.

Zhang, Z. (2016). Model building strategy for logistic regression: purposeful selection. Annals of translational medicine, 4(6), 1-7.

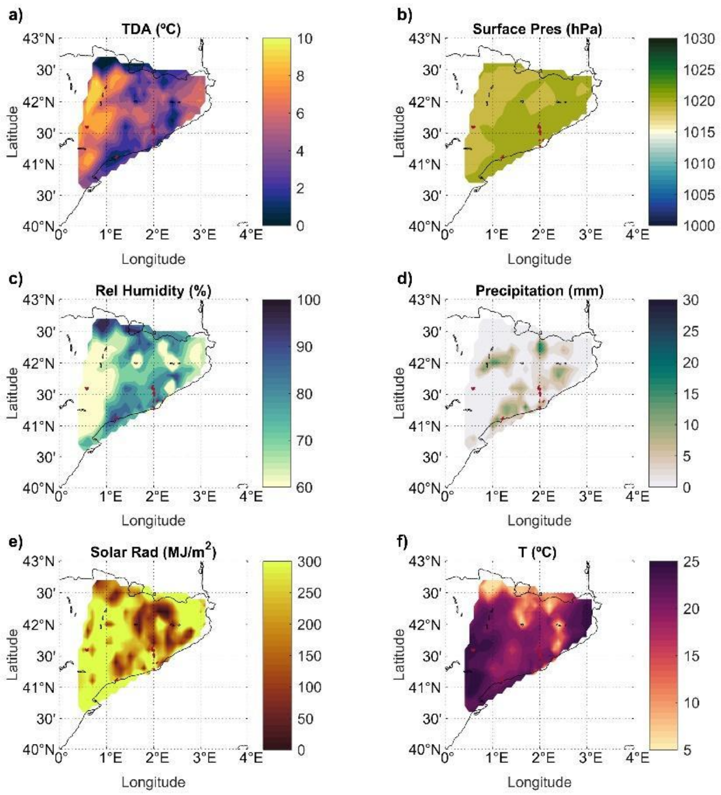

Figure S1. (a-f) Weather variables distribution along Catalonia on September 9, 2020, as obtained from the 187 automatic weather stations (AWS). The BHAs selected for our study are represented in red, with a population density $d \geq 500$ inhab km^{-2}.

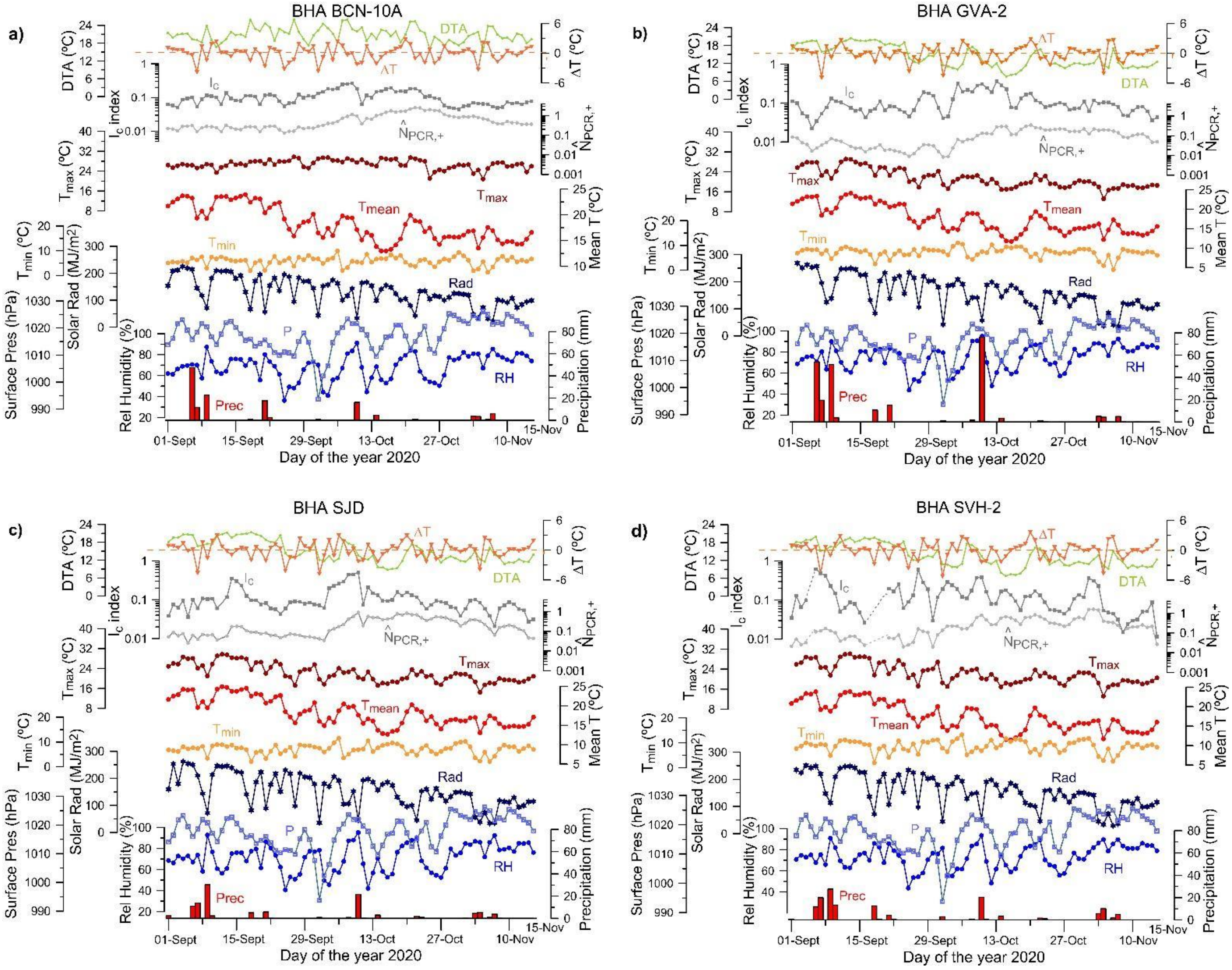

Figure S2. Time series of weather variables registered during the period that includes the COVID-19 second wave (September 1 to November 15, 2020) together with the normalized number of cases ($\widehat{N}_{PCR,+}$) and the infection rate (I_c) in all the BHAs, as indicated. The weather variables analysed in the study are daily mean temperature (T_{mean}), relative humidity (*RH*), solar radiation(*Rad*), precipitation (*Prec*), surface pressure (*P*), daily minimum (T_{min}) and maximum temperature (T_{max}), daily thermal amplitude (*DTA*) and difference in temperature between consecutive days (ΔT). The units for these variables are indicated in their corresponding axes.

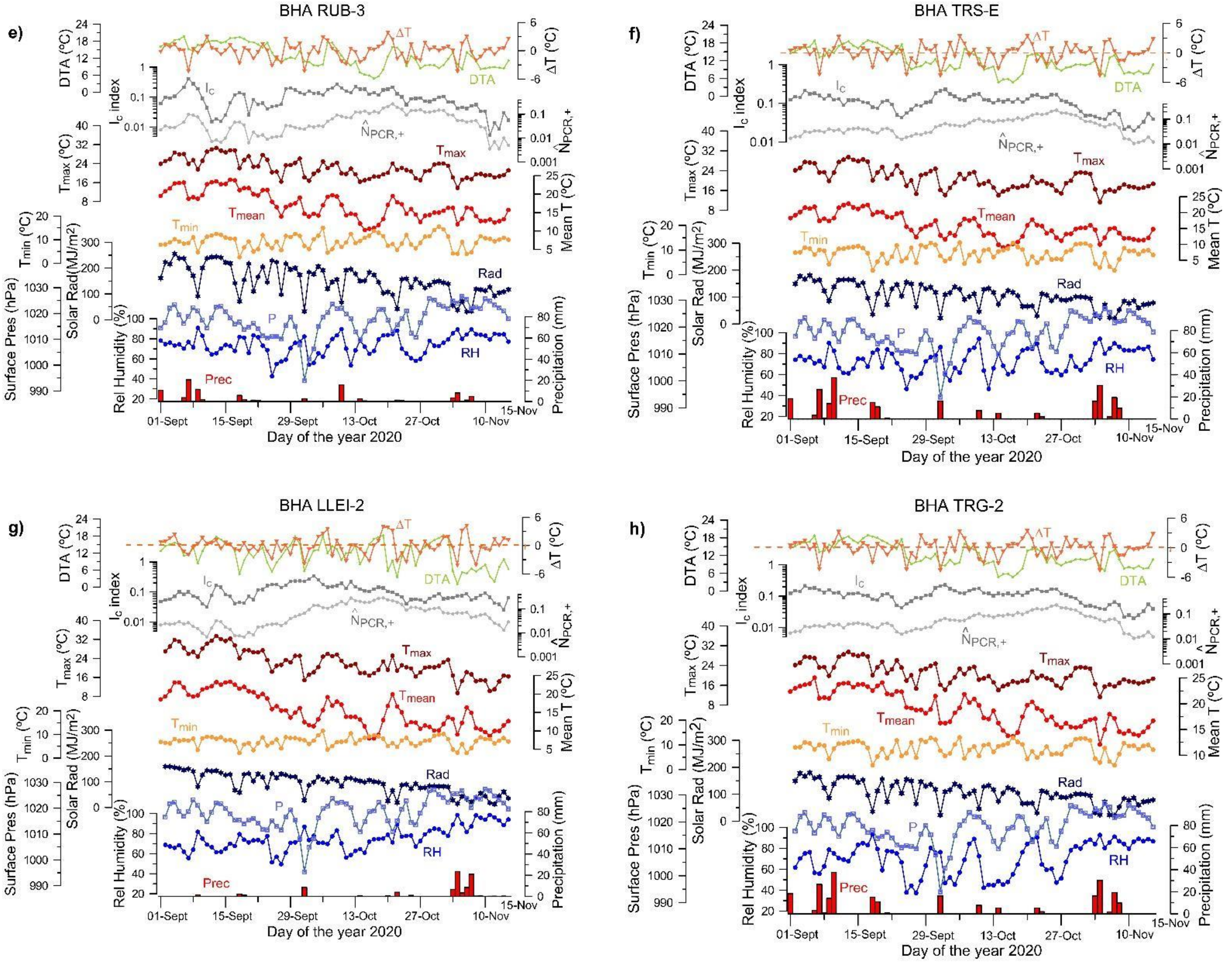

Figure S2 (continued)

a)

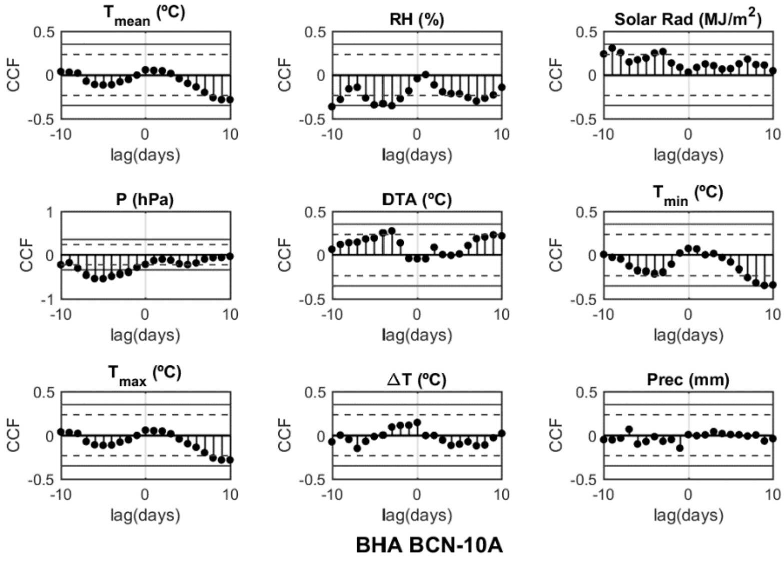

b)

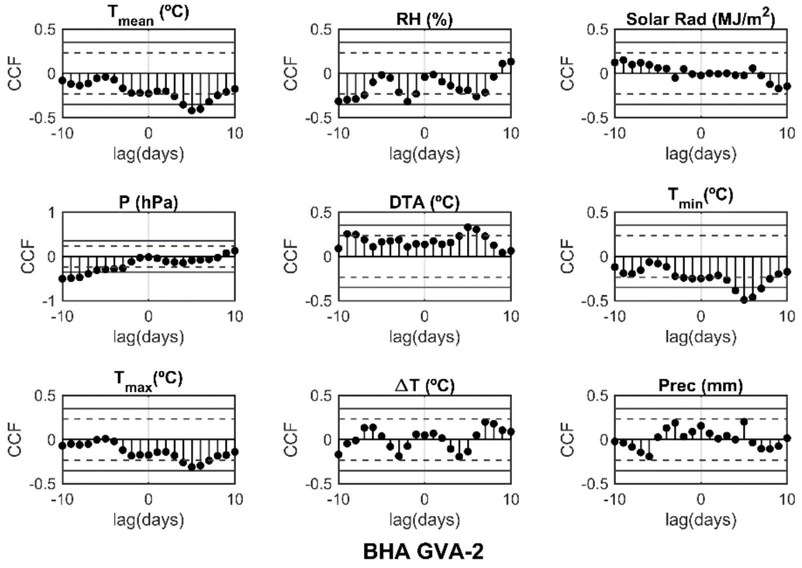

Figure S3. Cross-correlation coefficient functions of the infection index with the weather variables as a function of time lag, in days, for the eight BHAs: (a) BCN-10A, (b) GVA-2, (c) SJD, (d) SVH-2, (e) RUB-3, (f) TRS-E, (g) LLEI-2, and (h) TRG-2. The horizontal dashed and solid lines indicate the statistical significance at α=5% and α=1% .

c)

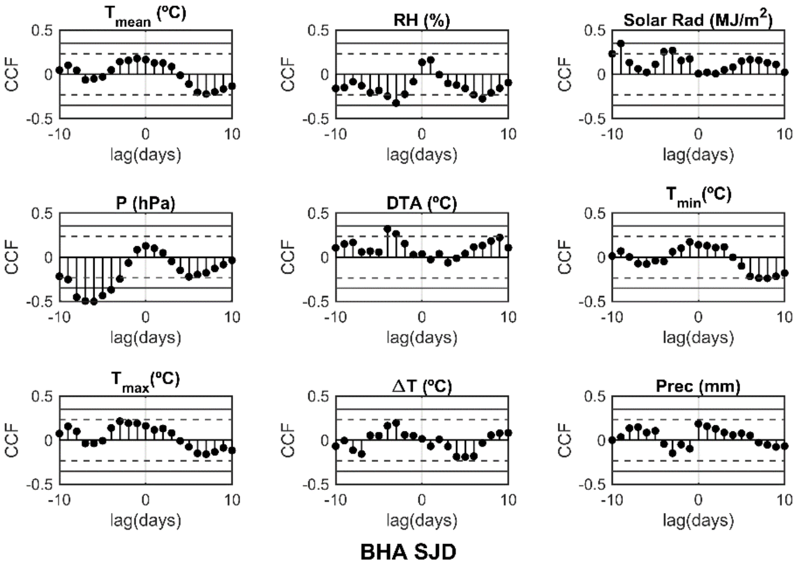

d)

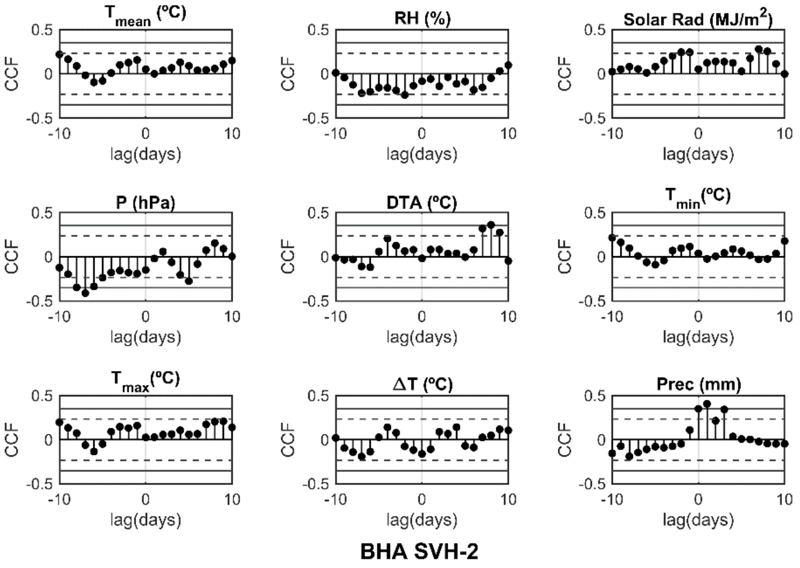

Figure S3 (continued)

e)

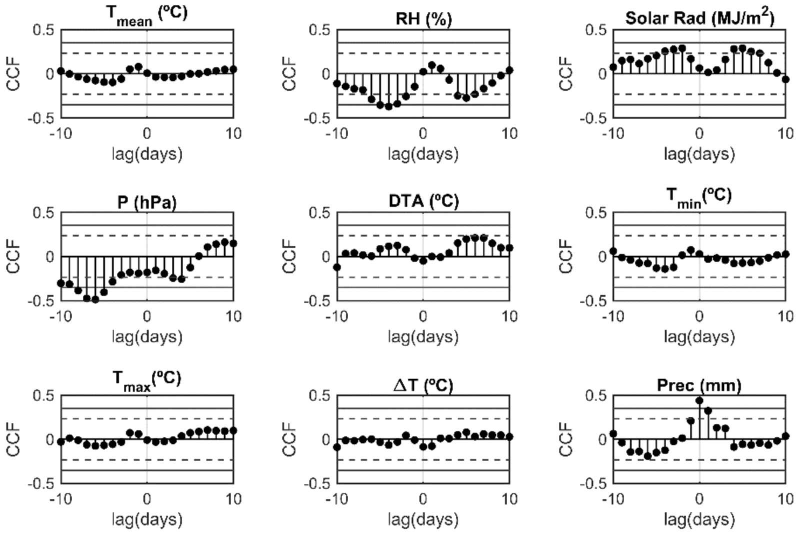

BHA RUB-3

f)

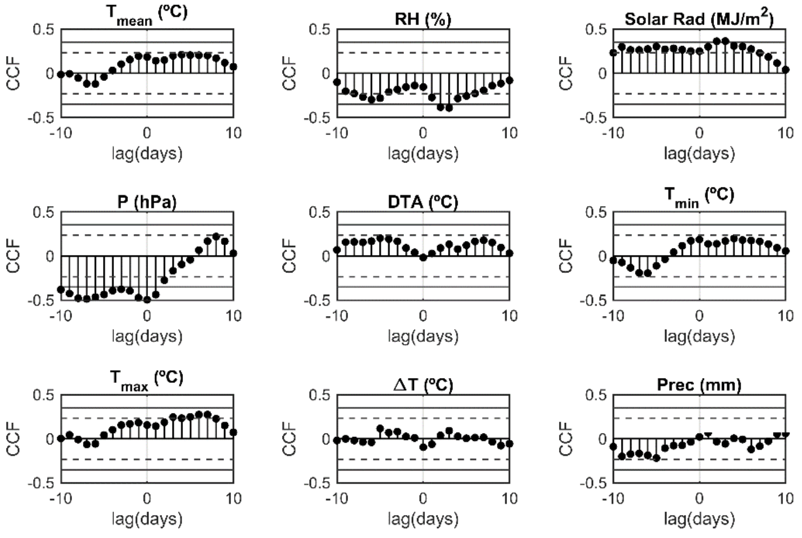

BHA TRS-E

g)

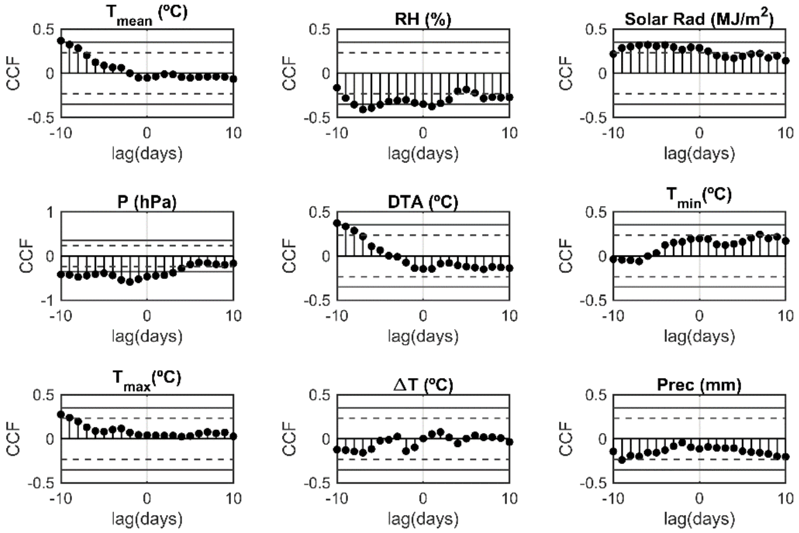

BHA LLEI-2

h)

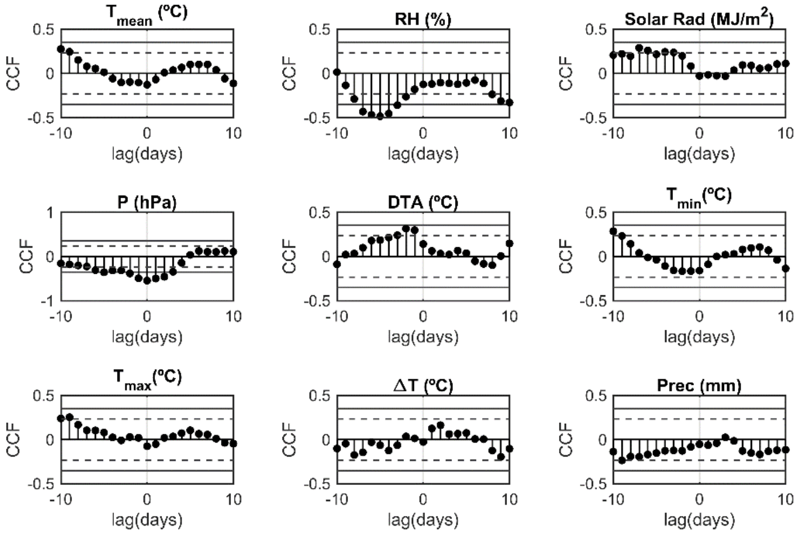

BHA TRG-2

Figure S3 (continued)

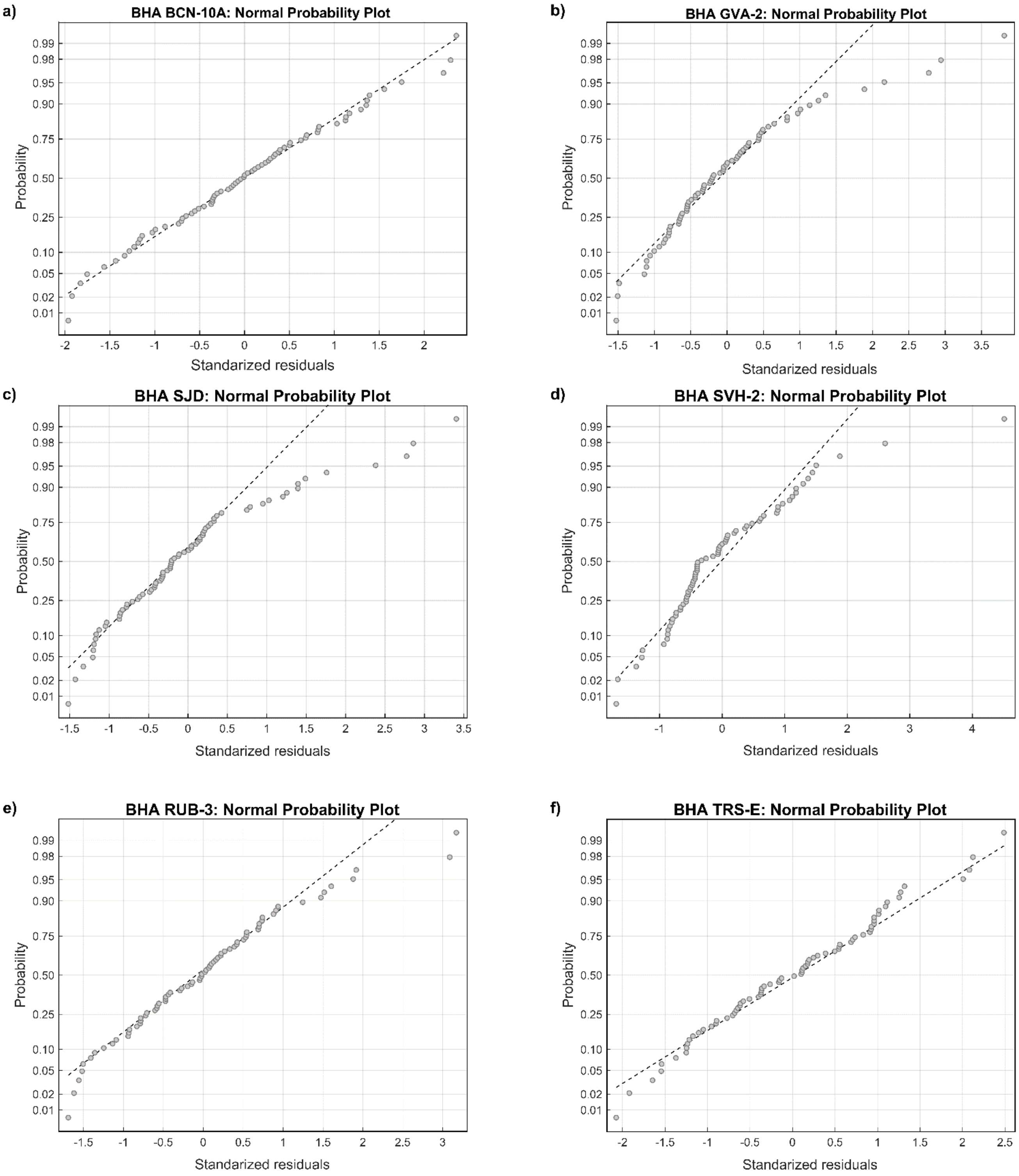

Figure S4. Probability plots of the standardized residuals, fitted to a normal distribution.

g)

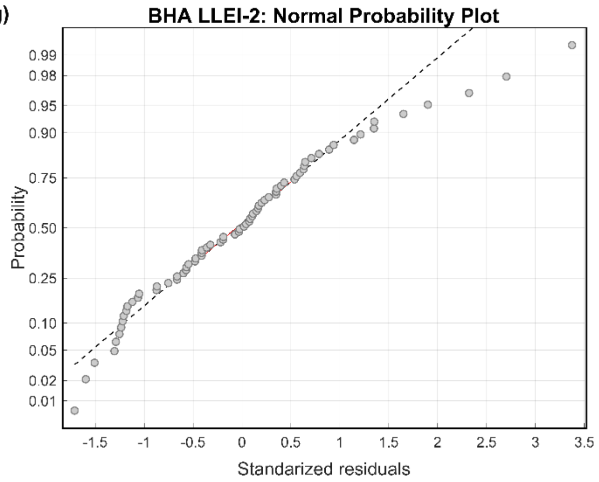

h)

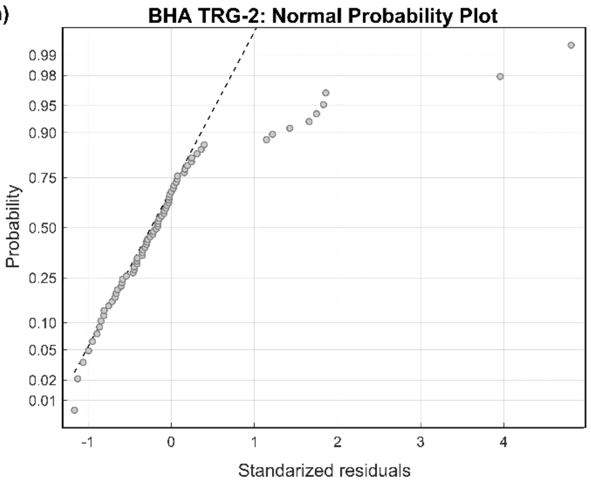

Figure S4 (continued)

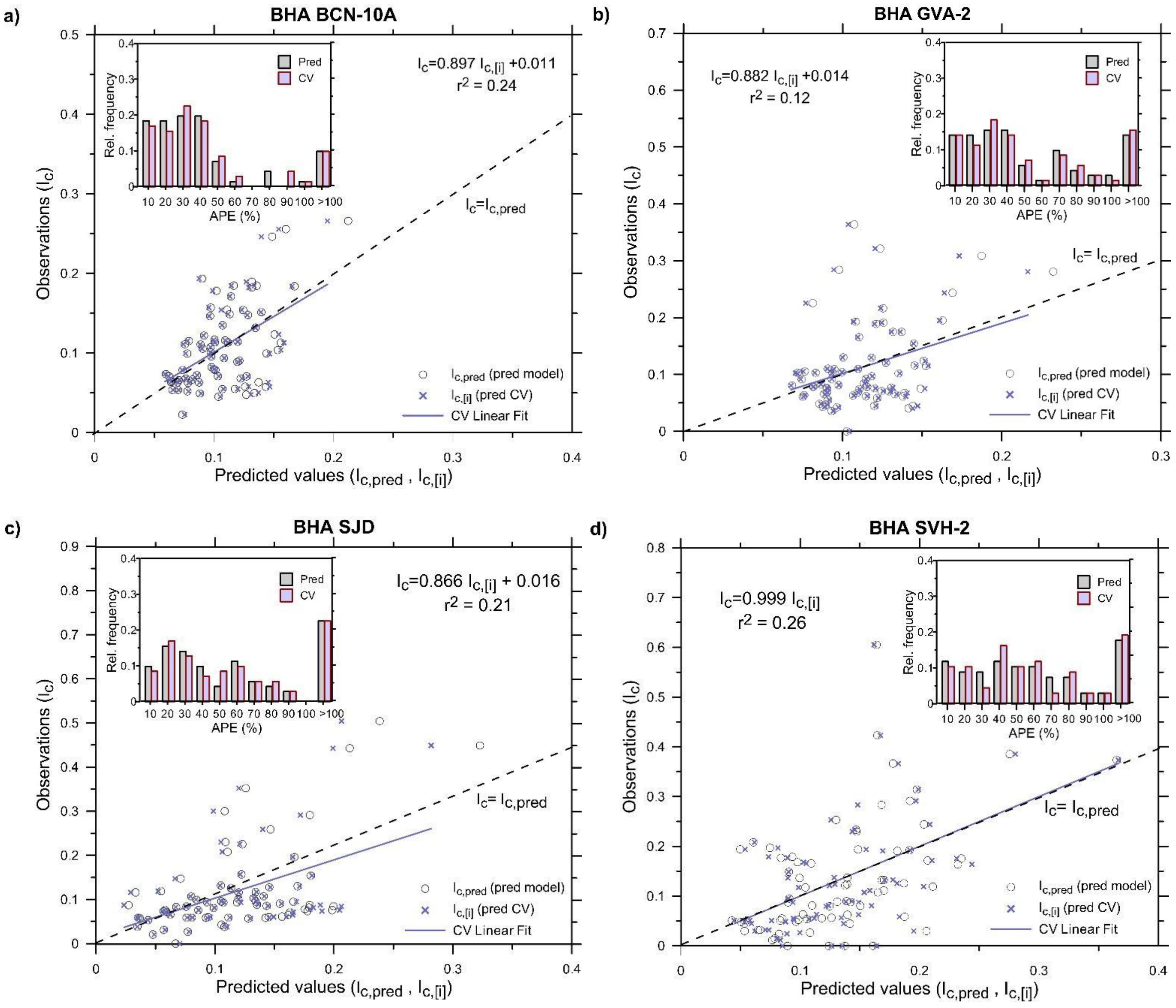

Figure S5. (a-h) Infection index I_c plotted as a function of the corresponding model predictions $I_{c,pred}$ (grey circles) and as a function of the cross-validated $I_{c,[i]}$ values obtained using the LOOCV method (blue crosses), plotted for all eight BHAs as labelled. The lines indicate the regression fits of the observations with the model predictions (dashed black lines) and the cross-validated data (blue solid lines). The linear equations that correspond to the cross-validation dataset (blue crosses) are displayed in each graph along with their R-squared. Each panel also includes a multiple bar chart of the absolute percentage errors (*APE*) as compared with the measurements, grouped at 10% intervals with the model (grey bars) and the cross-validation method (blue bars).

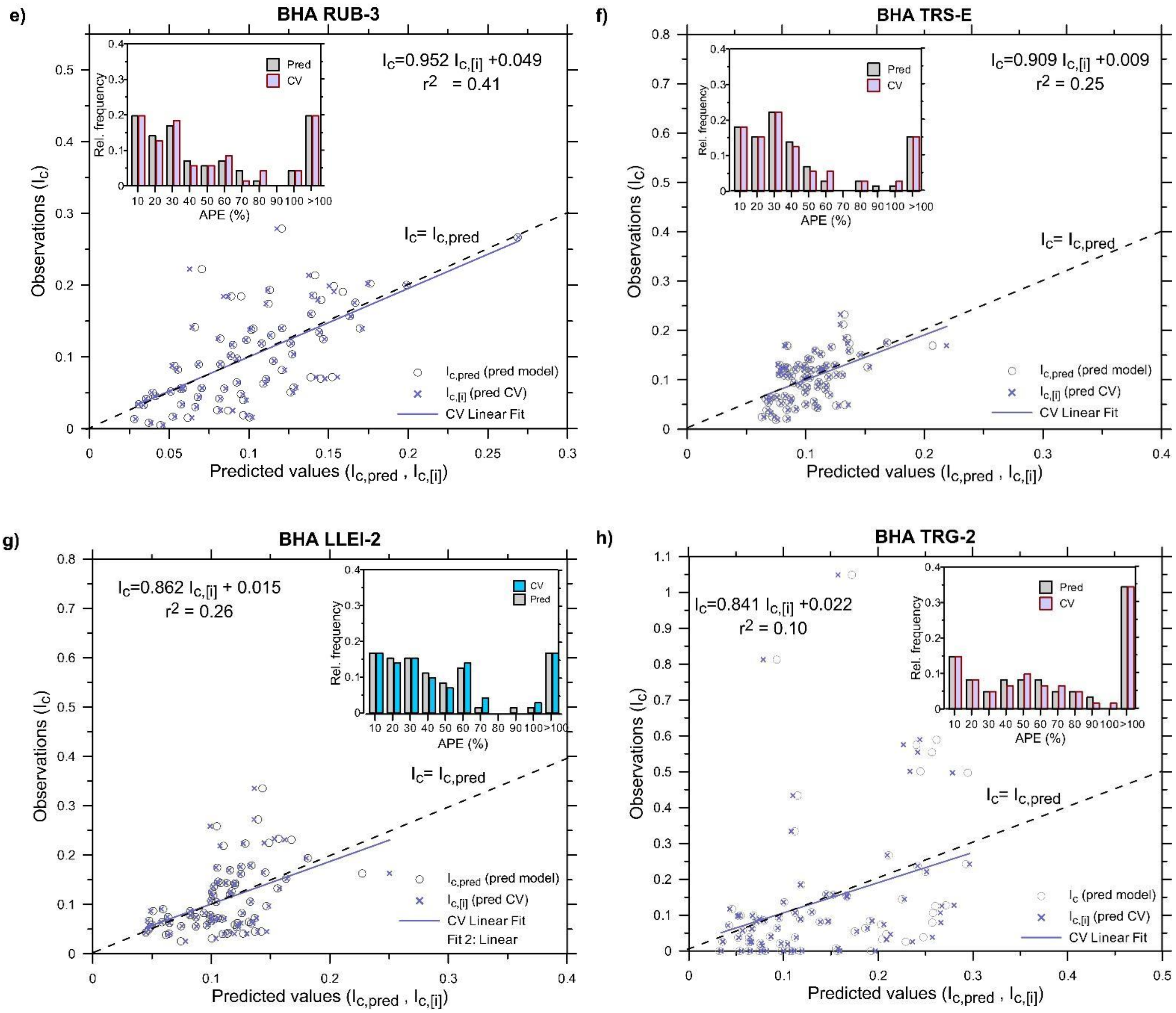

Figure S5 (continued)

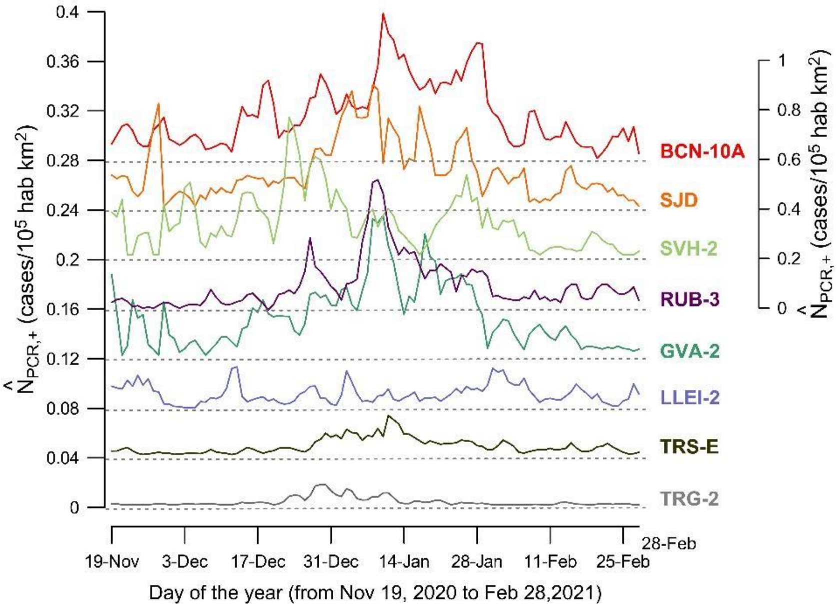

Figure S6. Temporal evolution of the number of COVID-19 cases in all BHAs from November 19, 2020, to February 28, 2021. The lower five time series (from TRG-2 to RUB-3) are represented against the left axis and the remaining BHAs (SVH-2, SJD and BCN-10A) against the right axis. The series are shifted to facilitate identifying the main features.

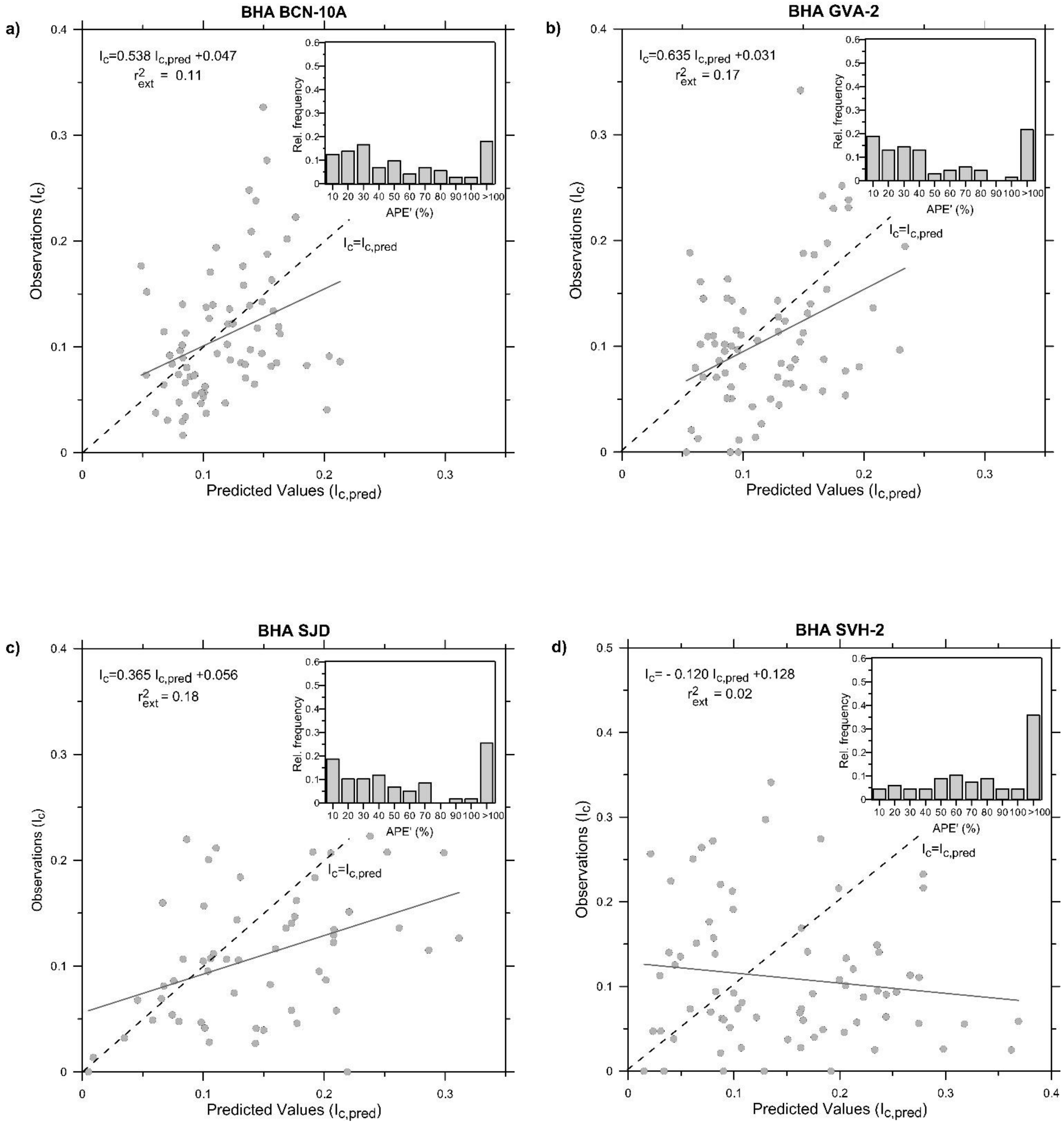

Figure S7. (a-h) Scatter plots of the infection index I_c as a function of the corresponding predictions $I_{c,pred}$ over the forecast period for each BHA. The dashed lines indicate the 1:1 relation and the solid lines represent the best-fit line between them. The linear equations of the regression fit are displayed in each graph along with their predicted R-squared, r^2_{ext}. The bar charts for the absolute prediction errors (*APE*') of the measurements, which have been grouped in intervals of 10%, are inserted in the upper part of each panel.

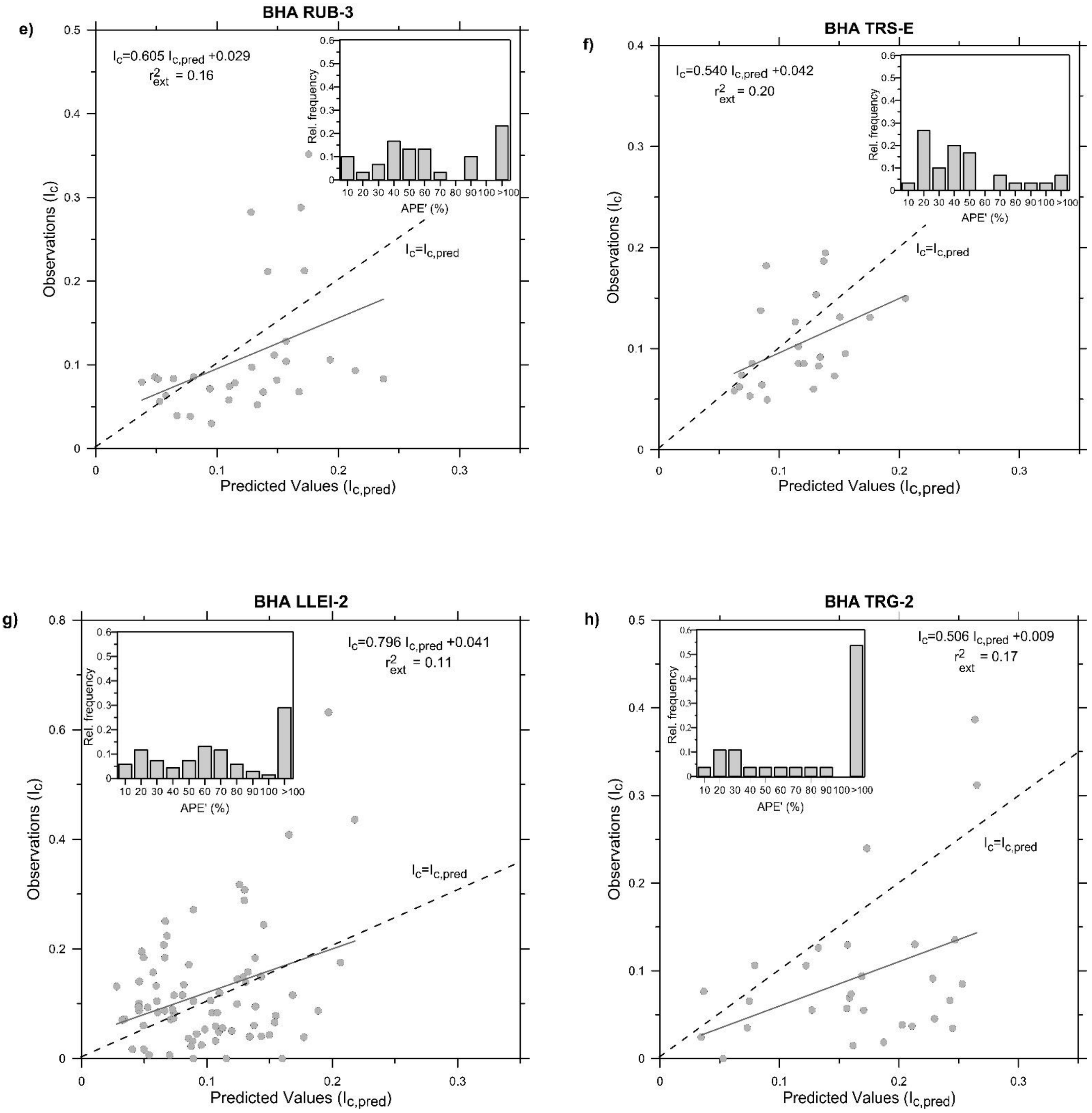

Figure S7 (continued)

	$r_{P,RH}$	t_{TS}	p_{TS}	*VIF*
**BCN-10A**	0.24^{**}	2.06	0.042	1.06
**GVA-2**	0.21^{*}	1.84	0.069	1.05
**SJD**	0.22^{*}	1.92	0.059	1.05
**SVH-2**	0.30^{**}	2.63	0.011	1.10
**RUB-3**	0.31^{***}	2.77	0.007	1.11
**TRS-E**	0.26^{**}	2.32	0.023	1.07
**LLEI-2**	0.36^{***}	3.29	0.002	1.15
**TRG-2**	0.30^{***}	2.66	0.005	1.10

Table S1. Statistical descriptors used to analyze the collinearity effects in the model: 1) $r_{P,RH}$ as the correlation coefficient between surface pressure and relative humidity; 2) t_{TS} as the *t*-statistic of the significance correlation test; 3) p_{TS} as the *p* value associated to the test statistic; 4) *VIF* as the variance inflation factor for the surface pressure. The asterisks '*' , '**' and '***' denote $p < 0.01$, $p < 0.05$ and $p < 0.1$, respectively, and the symbol '$^{+}$' indicates no statistical significance ($p \geq 0.1$).

	RMSE	r^2	r^2_{adj}	*F*-stat	*p*
**BCN-10A**	0.0445	0.31	0.29	15.7	< 0.01
**GVA-2**	0.0676	0.18	0.16	7.56	< 0.01
**SJD**	0.0827	0.31	0.29	15.3	< 0.01
**SVH-2**	0.0996	0.26	0.24	12.1	< 0.01
**RUB-3**	0.0505	0.45	0.43	28.0	< 0.01
**TRS-E**	0.0410	0.29	0.27	14.3	< 0.01
**LLEI-2**	0.0580	0.27	0.25	12.7	< 0.01
**TRG-2**	0.1830	0.17	0.14	6.82	< 0.01

Table S2. Goodness-of-fit parameters of the model as built with the two candidate predictors: surface pressure and relative humidity. Abbreviations: *RMSE*, root-mean-square error; r^2, the coefficient of determination; r^2_{adj}, the adjusted coefficient of determination; *F-stat*, the joint *F*-statistic, and *p* as its corresponding *p* value.

	c_0	*SE*	*t*-stat	*p*
**BCN-10A**	4.18^{***}	0.94	4.44	<0.01
**GVA-2**	4.98^{***}	1.44	3.47	<0.01
**SJD**	8.06^{***}	1.74	4.62	<0.01
**SVH-2**	9.83^{***}	2.14	4.60	<0.01
**RUB-3**	6.64^{***}	1.08	6.14	<0.01
**TRS-E**	4.42^{***}	0.86	5.13	<0.01
**LLEI-2**	5.22^{***}	1.31	-3.80	<0.01
**TRG-2**	5.83^{+}	4.05	1.44	0.15

Table S3. Statistical descriptors for the intercept c_0 of the relative humidity, in the model built with the two candidate predictors: surface pressure and relative humidity. Abbreviations: *SE,* the standard error of c_0; *t*-stat, the *t*-statistic of the hypothesis test; *p*, the *p* value of the hypothesis test. The asterisks '*' , '**' and '***' denote $p < 0.01$, $p < 0.05$ and $p < 0.1$, respectively, and the symbol '$^{+}$' indicates no statistical significance ($p \geq 0.1$).

	c_1 (10^{-3} hPa^{-1})	*SE* (10^{-3} hPa^{-1})	*t*-stat	*p*
**BCN-10A**	-3.92^{***}	0.93	-4.21	< 0.01
**GVA-2**	-4.71^{***}	1.42	-3.32	< 0.01
**SJD**	-7.69^{***}	1.72	-4.46	< 0.01
**SVH-2**	-9.49^{***}	2.12	-4.47	< 0.01
**RUB-3**	-6.31^{***}	1.07	-5.87	< 0.01
**TRS-E**	-4.22^{***}	0.85	-4.94	< 0.01
**LLEI-2**	-4.94^{***}	1.30	-3.80	< 0.01
**TRG-2**	-5.30^{+}	1.42	-2.88	0.19

Table S4. Statistical descriptors for the regression coefficient c_1 of the surface pressure, in the model built with the two candidate predictors: surface pressure and relative humidity. Abbreviations: *SE*, the standard error of c_1; t-stat, the t-statistic of the hypothesis test; *p*, the *p* value of the hypothesis test. The asterisks ‘*’ , ‘**’ and ‘***’ denote $p < 0.01$, $p < 0.05$ and $p < 0.1$, respectively, and the symbol ‘$^{+}$’ indicates no statistical significance ($p \geq 0.1$).

	c_2 (10^{-3})	*SE* (10^{-3})	*t*-stat	*p*
**BCN-10A**	-1.15^{**}	0.44	-2.57	0.012
**GVA-2**	-0.87^{+}	0.69	-1.26	0.211
**SJD**	-1.64^{**}	0.75	-2.19	0.032
**SVH-2**	-0.70^{+}	1.10	-0.63	0.529
**RUB-3**	-1.57^{**}	0.61	-2.58	0.012
**TRS-E**	-0.31^{+}	0.85	-4.94	0.497
**LLEI-2**	-1.07^{*}	0.63	-1.68	0.097
**TRG-2**	-4.11^{***}	1.42	-2.88	0.005

Table S5. Statistical descriptors for the regression coefficient c_2 of the relative humidity, in the model built with the two candidate predictors: surface pressure and relative humidity. Abbreviations: *SE*, the standard error of c_2; *t*-stat, the *t*-statistic of the hypothesis test; *p*, the *p* value of the hypothesis test. The asterisks ‘*’ , ‘**’ and ‘***’ denote $p < 0.01$, $p < 0.05$ and $p < 0.1$, respectively, and the symbol ‘$^{+}$’ indicates no statistical significance ($p \geq 0.1$).

	SSR_1	SSR_2	SSE_2	r_1^2	r_2^2	Δr^2	F_{par}	p
**BCN-10A**	0.0489	0.0620	0.1363	0.2464	0.3125	0.0661^{**}	6.60	0.012
**GVA-2**	0.0617	0.0690	0.3149	0.1608	0.1797	0.0189^{+}	1.58	0.213
**SJD**	0.2368	0.2027	0.4719	0.3067	0.2586	0.0481^{**}	4.79	0.032
**SVH-2**	0.2368	0.2407	0.6845	0.2559	0.2602	0.0043^{+}	0.40	0.529
**RUB-3**	0.1259	0.1428	0.1757	0.3953	0.4484	0.0531^{**}	6.65	0.012
**TRS-E**	0.0474	0.0482	0.1159	0.2887	0.2935	0.0048^{+}	0.47	0.495
**LLEI-2**	0.0758	0.0853	0.2320	0.2388	0.2687	0.0299^{*}	2.82	0.098
**TRG-2**	0.3977	0.4562	2.3075	0.1439	0.1651	0.0212^{+}	1.75	0.190

Table S6. Statistical parameters for the partial F-test. The subindexes 1 and 2 indicate the model with the remaining predictor removed (1) or added (2). Abbreviations: SSR, the sum of the squares of the regression for the corresponding model; SSE, the sum of the squares of the error for the corresponding model; r^2, R-squared for the corresponding model; Δr^2, difference between r_1^2 and r_2^2; F_{par}, partial F-statistic of the test; p, the p value associated with the test statistic. The critical value of the partial F-statistic at α = 10 % is $F_{par}^{*} = 2.78$. The asterisks '*', '**' and '***' denote $p < 0.01$, $p < 0.05$ and $p < 0.1$, respectively, and the symbol '$^{+}$' indicates no statistical significance ($p \geq 0.1$).

	c_0	c_1 (10^{-3} hPa^{-1})	c_2 (10^{-3})	*RMSE*	r^2	r^2_{adj}	*F*	*p*
**BCN-10A**	4.18^{***}	-3.92^{***}	-1.15^{**}	0.045	0.31	0.29	15.7	< 0.01
**GVA-2**	5.30^{***}	-5.106^{***}	0	0.068	0.16	0.15	13.4	< 0.01
**SJD**	8.06^{***}	-7.69^{***}	-1.64^{**}	0.083	0.31	0.29	15.3	< 0.01
**SVH-2**	10.20^{***}	-9.89^{***}	0	0.099	0.26	0.25	24.1	< 0.01
**RUB-3**	6.64^{***}	-6.31^{***}	-1.57^{**}	0.051	0.45	0.43	28.0	< 0.01
**TRS-E**	4.56^{***}	-4.38^{***}	0	0.041	0.29	0.28	28.4	< 0.01
**LLEI-2**	5.22^{***}	-4.94^{***}	-1.07^{**}	0.058	0.27	0.25	12.7	< 0.01
**TRG-2**	0.47^{***}	0	-4.67^{**}	0.184	0.14	0.13	11.8	< 0.01

Table S7. Final model parameters for the propagation of the virus in each BHA, as constructed by forward stepwise regression, with the intercept (c_0) and the two regression coefficients (c_1 and c_2). Other relevant statistical parameters: Root-mean-square error (*RMSE*) of the linear fit, the R-squared (r^2), its adjusted version r^2_{adj} and the joint *F*-statistic (*F*) along with its corresponding *p* value. The asterisks '*' , '**' and '***' denote $p < 0.01$, $p < 0.05$ and $p < 0.1$, respectively, and the symbol '$^+$' indicates no statistical significance ($p \geq 0.1$).

**Weather variable**	$\boldsymbol{c_i}$ (10^{-3})	***SE*** (10^{-3})	***p***
P	-4.50	0.94	<0.001
RH	-1.60	0.48	0.0015
Rad	-1.13	0.45	0.0140
DTA	-2.35	1.61	0.1450
T_{mean}	-1.14	1.90	0.5514
T_{min}	7.52	3.39	0.0293
T_{max}	-0.29	1.88	0.8763
DTA	-5.56	4.16	0.1854
Prec	-235.6	443.3	0.5967

Table S8. Univariable analysis of the weather variables for BCN 10-A. Abbreviations: c_i, the regression coefficient of the *i*-th weather variable ($\forall i \in [1,8]$); *SE*, the standard error of c_i ; *p*, the *p* value of the regression coefficient. The files highlighted in grey indicate a weather variable with *p* values of the regression coefficients greater than the cut-off value ($p > 0.25$).

**Weather variable**	$\boldsymbol{c_i}$ (10^{-3})	***SE*** (10^{-3})	***p***
P	-5.11	1.39	<0.001
RH	-1.37	0.72	0.0063
Rad	-0.13	0.14	0.3491
DTA	-4.59	2.37	0.0567
T_{mean}	-0.38	2.59	0.1479
T_{min}	6.12	4.38	0.1673
T_{max}	-0.35	2.48	0.1606
DTA	9.07	5.51	0.1043
Prec	53.92	32.89	0.1046

Table S9. Univariable analysis of the weather variables for GVA-2. Abbreviations: c_i, the regression coefficient of the i-th weather variable ($\forall i \in [1,8]$); *SE*, the standard error of c_i ; *p*, the *p* value of the regression coefficient. The files highlighted in grey indicate a weather variable with *p* values of the regression coefficients greater than the cut-off value ($p > 0.25$).

**Weather variable**	c_i (10^{-3})	***SE*** (10^{-3})	***p***
P	-8.47	1.72	<0.001
RH	-2.39	0.83	0.0050
Rad	-0.44	0.19	0.0207
DTA	-1.33	3.19	0.6772
T_{mean}	6.08	3.44	0.0821
T_{min}	1.49	5.62	0.7923
T_{max}	5.98	3.21	0.0664
DTA	11.28	6.71	0.0971
Prec	236.9	122.1	0.0565

Table S10. Univariable analysis of the weather variables for SJD. Abbreviations: c_i, the regression coefficient of the *i*-th weather variable ($\forall i \in [1,8]$); *SE*, the standard error of c_i ; *p*, the *p* value of the regression coefficient. The files highlighted in grey indicate a weather variable with *p* values of the regression coefficients greater than the cut-off value ($p > 0.25$).

**Weather variable**	c_i (10^{-3})	SE (10^{-3})	p
P	-9.89	2.02	<0.001
RH	-2.17	1.18	0.0071
Rad	-0.40	0.23	0.0791
DTA	-0.36	3.66	0.9229
T_{mean}	0.46	4.02	0.9095
T_{min}	2.69	5.57	0.6311
T_{max}	-0.13	3.67	0.9723
DTA	13.64	7.73	0.0818
$Prec$	184.4	138.8	0.1881

Table S11. Univariable analysis of the weather variables for SVH-2. Abbreviations: c_i, the regression coefficient of the i-th weather variable ($\forall i \in [1,8]$); SE, the standard error of c_i ; p, the p value of the regression coefficient. The files highlighted in grey indicate a weather variable with p values of the regression coefficients greater than the cut-off value ($p > 0.25$).

**Weather variable**	c_i (10^{-3})	*SE* (10^{-3})	***p***
P	-7.18	1.06	<0.001
RH	-2.69	0.70	0.0003
Rad	0.31	0.13	0.0228
DTA	-2.01	1.97	0.3124
T_{mean}	-0.38	2.13	0.8586
T_{min}	3.26	2.74	0.2381
T_{max}	-0.28	1.99	0.8902
DTA	3.26	4.69	0.4889
Prec	223.2	143.5	0.1244

Table S12. Univariable analysis of the weather variables for RUB-3. Abbreviations: c_i, the regression coefficient of the *i*-th weather variable ($\forall i \in [1,8]$); *SE*, the standard error of c_i ; *p*, the *p* value of the regression coefficient. The files highlighted in grey indicate a weather variable with *p* values of the regression coefficients greater than the cut-off value ($p > 0.25$).

**Weather variable**	$\boldsymbol{c_i}$ (10^{-3})	***SE*** (10^{-3})	***p***
P	-4.38	0.82	<0.001
RH	-0.90	0.50	0.0765
Rad	0.43	0.13	0.0011
DTA	-0.72	1.49	0.6295
T_{mean}	2.17	1.60	0.1792
T_{min}	3.06	1.95	0.1207
T_{max}	1.83	1.38	0.1920
DTA	2.15	1.95	0.2754
Prec	-143.6	74.6	0.0585

Table S13. Univariable analysis of the weather variables for TRS-E. Abbreviations: c_i, the regression coefficient of the i-th weather variable ($\forall i \in [1,8]$); *SE*, the standard error of c_i ; *p*, the *p* value of the regression coefficient. The files highlighted in grey indicate a weather variable with *p* values of the regression coefficients greater than the cut-off value ($p > 0.25$).

**Weather variable**	***c_i*** (10^{-3})	***SE*** (10^{-3})	***p***
P	-5.73	1.22	<0.001
RH	-1.94	0.64	0.0035
Rad	0.57	0.20	0.0053
DTA	-1.01	1.89	0.5924
T_{mean}	3.85	1.72	0.0288
T_{min}	-1.51	1.77	0.3980
T_{max}	2.26	1.51	0.1367
DTA	-5.35	4.16	0.2029
$Prec$	-144.4	95.2	0.1338

Table S14. Univariable analysis of the weather variables for LLEI-2. Abbreviations: c_i, the regression coefficient of the i-th weather variable ($\forall i \in [1,8]$); SE, the standard error of c_i ; p, the p value of the regression coefficient. The files highlighted in grey indicate a weather variable with p values of the regression coefficients greater than the cut-off value ($p > 0.25$).

**Weather variable**	$\boldsymbol{c_i}$ (10^{-3})	***SE*** (10^{-3})	***p***
P	-8.81	4.02	0.0317
RH	-0.65	1.28	<0.001
Rad	0.75	0.35	0.0390
DTA	-8.21	6.65	0.2213
T_{mean}	4.24	7.19	0.5570
T_{min}	28.1	1.02	0.0075
T_{max}	5.14	6.86	0.4560
DTA	2.15	1.95	0.2754
Prec	-818.4	543.4	0.1366

Table S15. Univariable analysis of the weather variables for TRG-2. Abbreviations: c_i, the regression coefficient of the i-th weather variable ($\forall i \in [1,8]$); *SE*, the standard error of c_i ; *p*, the *p* value of the regression coefficient. The files highlighted in grey indicate a weather variable with *p* values of the regression coefficients greater than the cut-off value ($p > 0.25$).

BHA	$c_{1,MLR}$ (10^{-3} hPa^{-1})	$c_{1,P\text{-}RH}$(10^{-3} hPa^{-1})	Δc_1(%)	$c_{2,MLR}$ (10^{-3})	$c_{2,P\text{-}RH}$(10^{-3})	Δc_2(%)
BCN-10A	-3.63	-3.92	8.0	-0.98	-1.15	17.3
SJD	-7.32	-7.69	5.1	-1.57	-1.64	4.5
RUB-3	-5.97	-6.31	5.7	-1.50	-1.57	4.7
LLEI-2	-5.34	-4.94	7.5	-1.09	-1.07	1.8
SVH-2	-8.50	-9.89	16.3	-	-	-
GVA-2	-6.15	-5.11	19.6	-	-	-
TRS-E	-4.08	-4.38	7.4	-	-	-
TRG-2	-	-	-	-5.71	-4.67	18.2

Table S16. Comparison between the two-step (MLR subindex) and the forward-step (P-RH subindex) methods used to build the model. The table includes the regression coefficients for surface pressure (c_1) and relative humidity (c_2), and the percent coefficient variation (Δc_i) between the two methods. The dash indicates that no comparison can be established as there is no regression coefficient for that variable in the forward stepwise model.

	KS-statistic	q_{KS}	χ^2	p_{HT}
**BCN-10A**	0.042	0.999	14.35	0.013
**GVA-2**	0.117	0.253	1.22	0.543
**SJD**	0.152	0.064	17.09	0.004
**SVH-2**	0.155	0.056	2.68	0.262
**RUB-3**	0.076	0.769	4.52	0.477
**TRS-E**	0.062	0.931	0.99	0.610
**LLEI-2**	0.078	0.739	0.05	0.022
**TRG-2**	0.236	< 0.001	2.21	0.331

Table S17. Results of the hypothesis tests conducted to inspect the assumptions of normality (Kolmogorov-Smirnov test) and heteroscedasticity (White test) of the residuals. The table includes the statistics (*KS* stat and χ^2 – stat) and the *p* values of each test.

	r^2	r^2_{adj}	*MAPE* (%)	*MAPE*	q^2_{CV}	$q^2_{CV,adj}$	$MAPE_{CV}$ (%)	$RMSE_{CV}$
**BCN-10A**	0.31***	0.29	40.02	0.044	0.24***	0.23	41.76	0.045
**GVA-2**	0.16***	0.15	50.50	0.067	0.12***	0.11	51.79	0.069
**SJD**	0.31***	0.29	59.24	0.082	0.21***	0.19	61.92	0.085
**SVH-2**	0.26***	0.25	79.76	0.098	0.26***	0.25	82.13	0.099
**RUB-3**	0.45***	0.43	75.85	0.050	0.41***	0.40	78.57	0.051
**TRS-E**	0.29***	0.28	48.90	0.040	0.25***	0.24	50.16	0.041
**LLEI-2**	0.27***	0.25	52.98	0.057	0.21***	0.20	55.19	0.058
**TRG-2**	0.14***	0.13	146.85	0.180	0.10***	0.09	150.73	0.185

Table S18. Summary of the statistics obtained from the datasets used to set the model and for cross-validation (CV), including the mean absolute prediction error (*MAPE*), the root-mean-square error (*RMSE*), the R-squared and its adjusted version. The asterisks '*' , '**' and '***' denote $p < 0.01$, $p < 0.05$ and $p < 0.1$, respectively, and the symbol '+' indicates no statistical significance ($p \geq 0.1$).

	q^2_{CV}	p (linear model)	β_1^{CV}	p (β_1^{CV}=1)	β_0^{CV}	p (β_0^{CV}=0)
**BCN-10A**	0.24***	< 0.01	0.897	0.59	0.011	0.53
**GVA-2**	0.12***	< 0.01	0.883	0.69	0.014	0.69
**SJD**	0.21***	< 0.01	0.866	0.51	0.016	0.52
**SVH-2**	0.26***	< 0.01	0.999	0.78	0	0.76
**RUB-3**	0.41***	< 0.01	0.953	0.73	0.005	0.75
**TRS-E**	0.25***	< 0.01	0.909	0.63	0.009	0.64
**LLEI-2**	0.21***	< 0.01	0.862	0.49	0.015	0.52
**TRG-2**	0.15***	< 0.01	0.844	0.52	0.022	0.56

Table S19. Summary of the results from the cross-validation (CV) linear fits, as done via the LOOCV method in each of the eight BHAs, including the R-squared (q^2_{CV}) of the cross-validation and the coefficients of the linear regression fit, β_1^{CV} (slope) and β_0^{CV} (y-intercept). The asterisks '*' , '**' and '***' denote $p < 0.01$, $p < 0.05$ and $p < 0.1$, respectively, and the symbol '+' indicates no statistical significance ($p \geq 0.1$).

	N	r^2_{ext}	p (linear model)	β^*_1	p (β^*_1=1)	β^*_0	p (β^*_0=0)
**BCN-10A**	72[a]	0.11^{***}	<0.01	0.538	0.020	0.047	0.04
**GVA-2**	72[a]	0.17^{***}	<0.01	0.635	0.012	0.014	0.15
**SJD**	56[b]	0.18^{***}	<0.01	0.365	< 0.01	0.056	< 0.01
**SVH-2**	72[a]	0.02^{+}	> 0.1	-0.120	n.a.	0.128	n.a.
**RUB-3**	30[c]	0.16^{**}	0.0284	0.605	0.14	0.029	0.16
**TRS-E**	24[d]	0.20^{**}	0.0288	0.540	0.05	0.042	0.15
**LLEI-2**	77[e]	0.11^{***}	<0.01	0.796	0.42	0.041	0.16
**TRG-2**	29[f]	0.17^{**}	0.0280	0.506	0.09	0.009	0.81

Table S20. Summary of the statistical parameters obtained for the external validation. N is the sample size and the superscript indicates the selected time intervals for the external validation: a, 19.11.2020 to 29.01.2021; b, 29.11.2020 to 23.01.2021; c, 31.12.2020 to 29.01.2021; d, 01.01.2021 to 24.01.2021; e, 19.11.2020 to 02.02.2021; f, 28.12.2020 to 25.01.2021. The statistical parameters are the predicted R-squared (r^2_{ext}), p and the β^*_1 (slope) and β^*_0 (y-intercept). The asterisks '*' , '**' and '***' denote $p < 0.01$, $p < 0.05$ and $p < 0.1$, respectively, and the symbol '$^+$' indicates no statistical significance ($p \geq 0.1$).

	r^2_{ext}	$r^2_{ext,adj}$	*MAPE* (%)	*RMSE*
**BCN-10A**	0.11^{***}	0.09	60.50	0.061
**GVA-2**	0.17^{***}	0.15	82.20	0.065
**SJD**	0.18^{***}	0.16	74.82	0.080
**SVH-2**	0.02^{+}	n.a	n.a	n.a.
**RUB-3**	0.16^{**}	0.14	69.43	0.075
**TRS-E**	0.20^{**}	0.16	38.32	0.044
**LLEI-2**	0.11^{***}	0.09	101.84	0.102
**TRG-2**	0.17^{**}	0.12	205.86	0.111

Table S21. Summary of the results of the regression fits for the external validation in each BHA. The table includes the predicted R-squared (r^2_{ext}), its adjusted version ($r^2_{ext,adj}$), the mean absolute prediction error (*MAPE*) and the root-mean-square error (*RMSE*). The asterisks '*' , '**' and '***' denote $p < 0.01$, $p < 0.05$ and $p < 0.1$, respectively, and the symbol '$^{+}$' indicates no statistical significance ($p \geq 0.1$).
